# Uses of NHANES Biomarker Data for Chemical Risk Assessment: Trends, Challenges, and Opportunities

**DOI:** 10.1289/ehp.1409177

**Published:** 2015-04-10

**Authors:** Jon R. Sobus, Robert S. DeWoskin, Yu-Mei Tan, Joachim D. Pleil, Martin Blake Phillips, Barbara Jane George, Krista Christensen, Dina M. Schreinemachers, Marc A. Williams, Elaine A. Cohen Hubal, Stephen W. Edwards

**Affiliations:** 1 National Exposure Research Laboratory, and; 2 National Center for Environmental Assessment, U.S. Environmental Protection Agency (EPA), Research Triangle Park, North Carolina, USA; 3 Oak Ridge Institute for Science and Education (ORISE) Participant, Research Triangle Park, North Carolina, USA; 4 National Health and Environmental Effects Research Laboratory, U.S. EPA, Research Triangle Park, North Carolina, USA; 5 National Center for Environmental Assessment, U.S. EPA, Washington, DC, USA; 6 National Health and Environmental Effects Research Laboratory, U.S. EPA, Chapel Hill, North Carolina, USA; 7 Office of Research and Development, U.S. EPA, Research Triangle Park, North Carolina, USA

## Abstract

**Background:**

Each year, the U.S. NHANES measures hundreds of chemical biomarkers in samples from thousands of study participants. These biomarker measurements are used to establish population reference ranges, track exposure trends, identify population subsets with elevated exposures, and prioritize research needs. There is now interest in further utilizing the NHANES data to inform chemical risk assessments.

**Objectives:**

This article highlights *a*) the extent to which U.S. NHANES chemical biomarker data have been evaluated, *b*) groups of chemicals that have been studied, *c*) data analysis approaches and challenges, and *d*) opportunities for using these data to inform risk assessments.

**Methods:**

A literature search (1999–2013) was performed to identify publications in which U.S. NHANES data were reported. Manual curation identified only the subset of publications that clearly utilized chemical biomarker data. This subset was evaluated for chemical groupings, data analysis approaches, and overall trends.

**Results:**

A small percentage of the sampled NHANES-related publications reported on chemical biomarkers (8% yearly average). Of 11 chemical groups, metals/metalloids were most frequently evaluated (49%), followed by pesticides (9%) and environmental phenols (7%). Studies of multiple chemical groups were also common (8%). Publications linking chemical biomarkers to health metrics have increased dramatically in recent years. New studies are addressing challenges related to NHANES data interpretation in health risk contexts.

**Conclusions:**

This article demonstrates growing use of NHANES chemical biomarker data in studies that can impact risk assessments. Best practices for analysis and interpretation must be defined and adopted to allow the full potential of NHANES to be realized.

**Citation:**

Sobus JR, DeWoskin RS, Tan YM, Pleil JD, Phillips MB, George BJ, Christensen K, Schreinemachers DM, Williams MA, Cohen Hubal EA, Edwards SW. 2015. Uses of NHANES biomarker data for chemical risk assessment: trends, challenges, and opportunities. Environ Health Perspect 123:919–927; http://dx.doi.org/10.1289/ehp.1409177

## Introduction

The Centers for Disease Control and Prevention (CDC) National Health and Nutrition Examination Survey (NHANES) is designed to assess the health and nutritional well-being of children and adults in the United States (CDC 2014a). Participation in NHANES is voluntary and confidential, and follows a complex, multistage, probability cluster design. Therefore, weighted NHANES data are considered representative of the entire U.S. (noninstitutionalized, civilian) population. Thousands of volunteers are invited each year to participate via interviews, questionnaires, and examinations. “Spot” biological samples (e.g., blood and urine at a single time point) are provided by many participants and analyzed for chemical biomarker levels. These biomarker data are published in the *National Reports on Human Exposure to Environmental Chemicals* (NER) stratified by age group, sex, and race/ethnicity (CDC 2014d). They are also made publically available online alongside demographic information, questionnaire responses, medical examination results, and other laboratory data (CDC 2014c).

The first NHANES survey (NHANES I) was conducted from 1971 to 1974 (CDC 2014b). NHANES II (1976–1980), Hispanic HANES (1982–1984), and NHANES III (1988–1994) preceded what is now a continuous survey (1999–present). NHANES II was the first to evaluate biomarkers of environmental chemical exposure—specifically, blood lead levels. Chemical biomonitoring was expanded in NHANES III to include biomarkers of selected pesticides, phthalates, and volatile organic compounds (VOCs). The number of monitored chemical biomarkers rose from 27, as captured in the first NER (data for 1999; CDC 2001), to 116 in the second NER (data for 1999–2000; CDC 2003), 148 in the third NER (data for 2001–2002; CDC 2005), and 212 in the most recent (fourth) NER (data for 2003–2004; CDC 2009). The February 2015 “Updated Tables” of the fourth NER include additional biomonitoring data for NHANES 2005–2006, 2007–2008, 2009–2010, and 2011–2012, bringing the current total to 265 chemical biomarkers (CDC 2015b). This most current suite of biomarkers incorporates analytes from more than a dozen chemical groups, including brominated flame retardants (BFRs), dioxins and furans, environmental phenols, fungicides, herbicides, insecticides [e.g., organophosphates (OPs), organochlorines (OCs), pyrethroids, carbamates], metals/metalloids, perfluorinated compounds (PFCs), phthalates, polychlorinated biphenyls (PCBs), polycyclic aromatic hydrocarbons (PAHs), VOCs, and others.

The U.S. Environmental Protection Agency (EPA) has used NHANES chemical biomarker data to support various research and regulatory activities, most notably the decision to remove lead from gasoline (U.S. EPA 1986, 2003). Yet, the U.S. Government Accountability Office (GAO) reported in 2009 that the U.S. EPA “has made limited use of biomonitoring data in its assessments of risk posed by commercial chemicals” (GAO 2009). The GAO recommended that the U.S. EPA develop a strategy to categorize existing biomonitoring data, identify limitations in analytic approaches, and prioritize data gaps. The National Research Council (NRC) of the National Academies has also recommended the increased use of biomarker data to support risk assessment activities (NRC 2006, 2007, 2009). In their 2012 publication *Exposure Science in the 21st Century: A Vision and a Strategy*, the NRC reported that “The NHANES data provide a unique and growing potential for evaluating source–exposure and exposure–disease relationships in a national population-based representative sample” and that biomarker data sets “will be essential for evaluating the efficacy of exposure reduction policies, and for prioritizing and assessing chemical risks” (NRC 2012). In response to these reports, we examined NHANES-related publications over the past 15 years (1999–2013) for the purpose of highlighting specific uses of the chemical biomarker data. Attention is given to the percentage of NHANES-related publications that have focused on chemical biomarkers and to the chemical groups that have been commonly studied. To identify the potential for impacts on risk assessment activities, publications were examined for their approaches to assessing chemical exposures and to linking exposures to measures of human health. Consistent with the GAO recommendations, the goals of this study were to highlight the state of the science for interpreting NHANES chemical biomarker data, challenges that can limit the use of these data in risk assessments, and opportunities to enhance data interpretation strategies.

## Methods

Publications that have reported on U.S. NHANES data were identified using the PubMed Advanced Search Builder (http://www.ncbi.nlm.nih.gov/pubmed/advanced). The PubMed search was performed in two steps (specific search strings are provided in Supplemental Material, Table S1). For step one, publications from 1999–2013 that included query terms for “NHANES” (or “National Health and Nutrition Examination Survey”) and “United States” (or “U.S.A.,” “USA,” “U.S.,” or “US”) in the title/abstract were identified. Query terms for “United States” were included because publications based on non-U.S. NHANES data (e.g., Korea NHANES) were identified in preliminary test searches. For step two, additional query terms related to biomarkers (i.e., “biomarker,” “biomarkers,” “biomonitoring,” “urine,” “urinary,” “blood,” and “serum”) were added. Search results from steps one and two were separated by publication year using a PubMed filter.

Publications identified in step two of the literature search were manually curated using published titles and abstracts. Publications were selected for additional analysis only if they clearly utilized NHANES chemical biomarker data. For this investigation, “chemical biomarkers” did not include endogenous biomarkers (e.g., hormones, antibodies, inflammatory markers), tobacco-specific biomarkers (e.g., cotinine), dietary biomarkers (e.g., vitamins/nutrients, essential minerals), or biomarkers of phytoestrogens, isoflavonoids, or aflatoxin. If a study’s use of NHANES chemical biomarker data could not be determined using only the published title and abstract, the full text was obtained and examined to inform the final selection decision.

During the manual curation, it was determined for selected publications which specific chemical biomarkers were studied and which analysis approaches were used (see Supplemental Material, Table S2). Decisions regarding chemical biomarker groupings and analysis approaches for all publications were made by a single author (J.R.S.) followed by a review of each classification by one of the other authors. Specific chemical biomarkers were first organized into chemical groups using guidance from NHANES documents (e.g., CDC 2015a). Certain chemical groups were then combined to allow a streamlined trends analysis. For example, dioxins, furans, and PCBs were considered as a single group, as were insecticides, herbicides, and fungicides (termed “pesticides”). Finally, each publication was assigned to one of the designated chemical groups. Studies that reported on at least two of the defined chemical groups were considered “multi-group.”

Selected publications were also assigned to one of two primary data analysis categories, defined here as “exposure assessment” and “health association”; studies in both analysis categories are considered relevant to the risk assessment process. Health association studies examined statistical associations between chemical biomarker levels and health measures (e.g., disease status, medical examination results). Exposure assessment studies were broadly defined and used chemical biomarker data to *a*) establish reference ranges for the U.S. population, *b*) evaluate data from other (non-NHANES) studies, *c*) track exposure trends over time, *d*) evaluate differences in exposure across population subsets, *e*) identify important predictors of exposure, or *f* ) estimate the percentage of the population with exposures that exceed a reference level. Many exposure assessment studies performed a combination of these analyses. Thus, it was not feasible to partition these studies into smaller categories. Investigations that addressed both exposures and health associations were categorized as health association studies.

Data were analyzed using Microsoft Excel (Office 2013; Microsoft Corporation, Redmond, WA) and SAS statistical software (v. 9.3; SAS Institute Inc., Cary, NC). Figures were prepared using Microsoft Excel, GraphPad Prism (v. 4.03, GraphPad Software, San Diego, CA), and R (v. 3.0.1) (R Core Team 2013).

## Results

**Figure 1:**
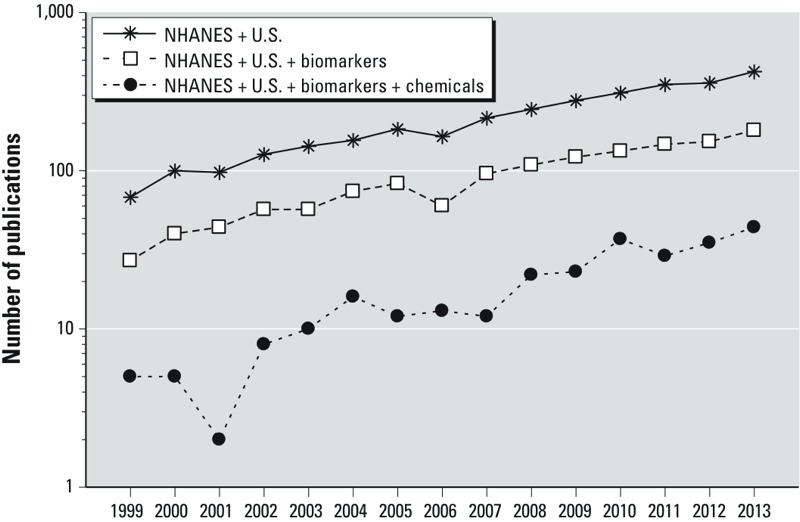
Yearly publications (1999–2013) related to the U.S. NHANES, biomarkers, and biomarkers of environmental chemicals. For PubMed search and selection methods, see Supplemental Material, Table S1.

*Yearly publications*. Sixty-eight publications from 1999 were identified that contained keywords related to “NHANES” and “United States” (Figure 1). More than 400 publications were identified from 2013 using the same search criteria. These results of sampled publications reflect a 6-fold increase over a 15-year span, and a median yearly increase of 13%. Considerably fewer publications were identified after adding additional keywords related to “biomarkers.” Only 27 publications from 1999 were identified that contained keywords related to “NHANES,” “United States,” and “biomarkers.” Close to 200 publications from 2013 were identified using the same keywords, indicating an approximate 7-fold increase over the 1999 baseline. Interestingly, the yearly ratios of biomarker-related publications (step two results) to total NHANES-related publications (step one results) were fairly consistent, ranging from 0.36 to 0.47, with a median value of 0.43. Results from a simple regression analysis showed no significant linear trend (*p* = 0.7) in this ratio, suggesting that the proportional use of NHANES biomarker data (not specific to chemical biomarkers) has been stable over the period of time examined in this study.

Only a small percentage of the total sampled NHANES-related publications specifically reported on chemical biomarkers (8% yearly average). The number of identified publications elevated from 5 in 1999 to 44 in 2013, representing a 9-fold increase over 15 years. The yearly ratios of chemical biomarker–related publications (manual curation results) to total NHANES-related publications (step one results) increased from 0.07 in 1999 to 0.10 in 2013. Simple linear regression results showed a significant positive effect (*p* = 0.007) of publication year on ratio estimates. This result suggests an increase over time in the proportion of NHANES-related studies that focus on chemical biomarker measurements.

**Figure 2:**
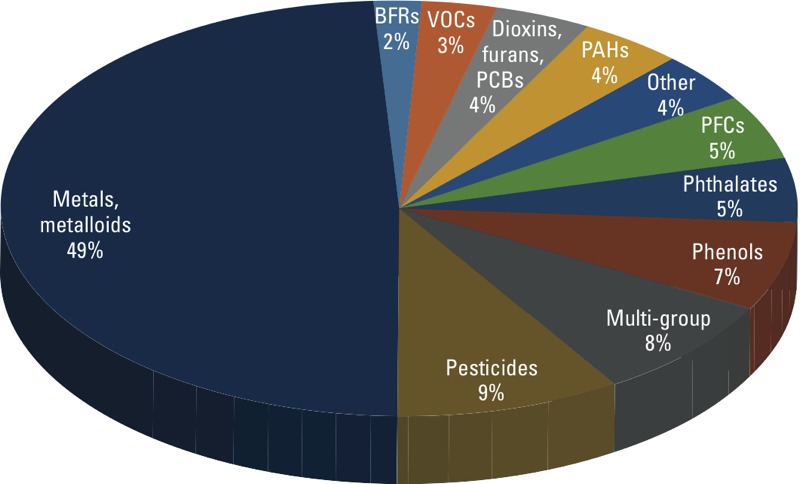
Chemical groups studied using NHANES biomarker data.

*Chemical groups*. Each publication identified through manual curation was assigned to 1 of 11 groups based on the chemical biomarkers that were studied (Figure 2). Metals/metalloids were by far the most commonly studied group. Studies of metals/metalloids (particularly lead, cadmium, mercury, and arsenic) comprised nearly half (49%) of the chemical biomarker–related publications. The second most studied chemical group was pesticides (9%), which included OP, OC, and pyrethroid insecticides, as well as herbicides, fungicides, and halogenated phenolic compounds. Environmental phenols (including bisphenol A, triclosan, and parabens) were the third most studied group (7%), followed by phthalates (5%), PFCs (5%), PAHs (4%), dioxins/furans/PCBs (4%), VOCs (3%), and BFRs (2%). Multi-group studies comprised 8% of the chemical biomarker–related publications. The remaining 4% of studies focused on a group defined as “other” chemicals; 7 of the 10 publications in this group focused on perchlorate.

**Figure 3:**
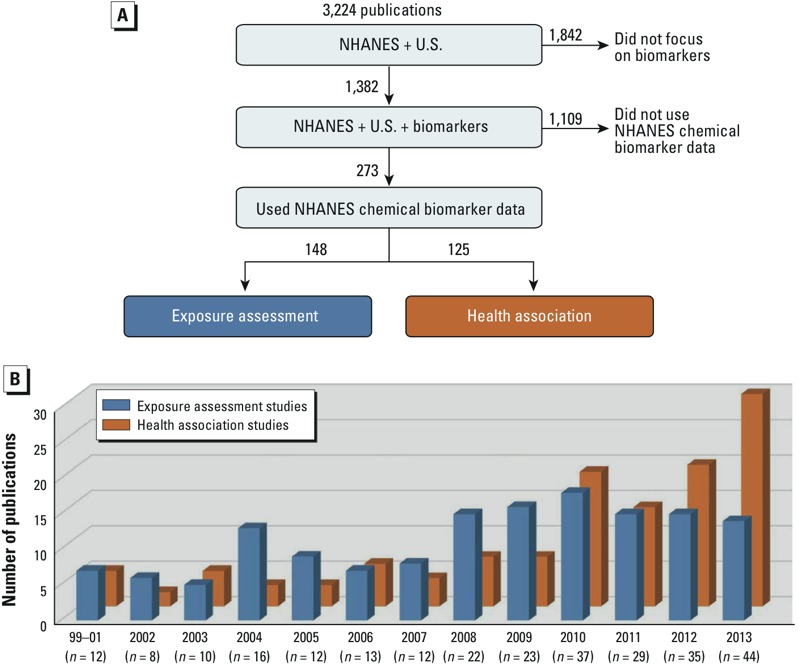
Tree diagram of publications identified via PubMed searches, selected via manual curation, and categorized by a data analysis approach (*A*). Trends in data analysis approaches for 1999–2013 (*B*).

*Analysis categories*. Step one of the PubMed literature search (NHANES + U.S. query) yielded 3,224 publications, step two (NHANES + U.S. + biomarkers query) yielded 1,382 publications, and manual curation yielded 273 publications (Figure 3A). Of the 273 studies that focused on chemical biomarkers, 148 (54%) performed an exposure assessment and 125 (46%) examined health associations. These results suggest that the chemical biomarker–related publications are evenly split between analysis categories over the past 15 years. Figure 3B shows the number of yearly publications for the two analysis categories. Limited numbers of papers were observed early in the review period, so data across 1999, 2000, and 2001 were combined. No trends were observed for either category prior to 2008. However, a sharp rise in exposure assessment studies was observed for 2004, and then again for 2008. These elevations likely reflect releases of the NHANES 1999–2000, 2001–2002, and 2003–2004 data sets (CDC 2003, 2005, 2009). The number of yearly exposure assessment studies remained relatively flat between 2008 and 2013. Health association studies, however, increased dramatically in number over the last 5 years of the review period. In fact, nearly 70% of the curated 2013 publications focused on health associations. This suggests growing interest in using the NHANES data to link chemical biomarkers and health measures.

**Figure 4:**
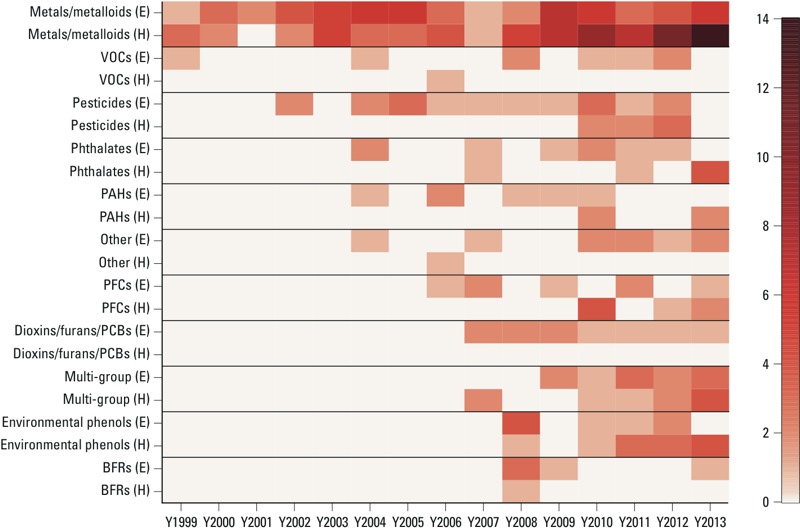
Yearly chemical biomarker–related publications stratified by chemical group and analysis category. Abbreviations: E, exposure assessment; H, health association. Darker colors reflect a higher number of publications for the specified chemical group in a particular year. The scale indicates the publication count by color.

*Trends by group and category*. The number of yearly chemical biomarker–related publications, after stratification by chemical group and analysis category, are shown in Figure 4. Between 1999 and 2003, publications focused almost exclusively on metals/metalloids (28 of 31); the strong focus on this group continued across all 15 years of the review period. A lack of publications related to other chemical groups prior to 2004 mirrors the public release dates of the NERs; although data on metals and select VOCs, pesticides, and phthalates were available in 1999 (from NHANES III), data on additional chemicals were not available until later years (CDC 2003, 2005, 2009). Indeed, Figure 4 illustrates that initial studies involving PAHs were published in 2004, and those involving PFCs, dioxins/furans/PCBs, environmental phenols, and BFRs were published between 2006 and 2008.

Exposure assessment studies preceded health association studies for most chemical groups (Figure 4). This is not surprising, given that many early studies focused on establishing biomarker reference ranges for the U.S. population (Barr et al. 2004; Calafat et al. 2008a, 2008b; Grainger et al. 2006; Nichols et al. 2007; Silva et al. 2004; Sjödin et al. 2008). For dioxins/furans/PCBs, VOCs, BFRs, pesticides, and “other” chemicals, exposure assessment studies comprised the majority of the group-specific publications (> 70% in each case). The number of publications was more balanced across analysis categories for metals/metalloids, phthalates, PFCs, environmental phenols, and multi-group chemicals. For these groups, between 40% and 60% of the publications focused on exposure assessment. The recent upward trend in health association studies (Figure 3B) is reflected most clearly for metals/metalloids, environmental phenols, and multi-group chemicals (Figure 4). An increasing focus on exposure assessments of multi-group chemicals is also evident over recent years (Figure 4).

## Discussion

NHANES is one of the largest continuous sources of chemical biomarker data in the United States. The publications included in this review have collectively reported on tens of thousands of measurements from representative samples of the U.S. population. However, it appears that a fairly small percentage of published studies related to NHANES have focused on chemical biomarker data. Indeed, for most years in our review period (1999–2013), < 10% of the total sampled NHANES-related publications focused on these data. It is difficult to know the expected or optimal use of these data as a fraction of total NHANES-based studies. The results shown here should therefore be considered as a point of reference for future evaluations of NHANES data usage. Assuming continued support for the NHANES programs, it is expected that the focus on chemical biomarkers will continue to increase as more scientists become aware of potential uses of the data. Specifically, interest will rise, given the availability of new methods and applications for interpreting NHANES data in health risk contexts. Below, we discuss key findings of this review, challenges related to these findings that can limit the use of NHANES data for chemical risk assessment, and examples of new methods and guidance that will help future studies overcome these challenges.

*Key findings*. According to our sample of publications, biomarkers of metals/metalloids have been studied far more frequently than those of other chemical groups (Figures 2 and 4). There are several reasons for this imbalance. First, biomarker levels of select metals have been reported for a broader participant age range (including children younger than 6 years of age), and over more survey years. For example, blood lead was monitored in NHANES II (1976–1980), whereas biomonitoring for environmental phenols, PFCs, and BFRs began in NHANES 2003–2004. A second reason is that biomarker-based reference levels exist for certain metals (e.g., lead, mercury) based on empirical biomarker–response relationships from epidemiological studies. These reference levels allow direct risk-based interpretation of the NHANES measurements. For most chemicals, however, biomarker reference levels are not available, thereby limiting the direct use of biomarker data in this context. For these chemicals, models are required to link biomarker measurements to external exposure reference levels (described in detail below), such as a U.S. EPA reference dose (RfD).

Despite the availability of biomarker data for hundreds of other chemicals, the number of publications on metals/metalloids appears to have increased over the past 5 years [particularly studies of health associations (Figure 4)]. However, it is also apparent that the focus is beginning to broaden across chemical groups. In particular, there is evidence for increasing attention on both multi-group exposure assessment and health association studies (Figure 4). Studies of this nature will be necessary to systematically evaluate impacts of multiple chemical stressors on human health. It is important to note, however, that these multi-group studies are restricted to the inventory of chemical biomarkers in NHANES and therefore still represent targeted assessments (Pleil and Stiegel 2013). Also, measurements of the full biomarker panel are not available for all study participants. Rather, select chemical biomarkers are measured in different subsamples, often due to limited volumes of collected blood and urine. Although NHANES biomarkers may be grouped based on common exposure sources and/or health end points, a lack of complete concordance challenges comprehensive evaluations of chemical mixtures. Finally, it must be understood that the totality of human exposures, defined as the “exposome,” is not restricted to exogenous chemical pollutants, but includes stressors from diet, drugs, infections, radiation, endogenous processes, and so on (Rappaport 2011; Rappaport and Smith 2010; Wild 2005). A wealth of information related to these stressors is now captured in NHANES questionnaires. Thus, researchers are encouraged to embrace the concept of the exposome when evaluating NHANES data and use data-driven approaches for the simultaneous evaluation of chemical and nonchemical stressors. Ultimately, nontargeted analyses of biological specimens, capturing stressor and response molecules, can supplement targeted NHANES measures and, together, allow discoveries of broader associations between exposures and health (Rappaport 2012; Rappaport et al. 2014).

Over the entire 15-year review period, the number of publications was fairly balanced across analysis categories, with about half focused on exposure assessment and half on health associations. A surprising result was the recent dramatic increase in published health association studies (Figure 3B). Results of our literature search (see Supplemental Material, Table S2) suggest that these types of studies have been performed using biomarkers across nearly all NHANES chemical groups. Furthermore, our results indicate that individual chemical biomarkers have been examined for associations with a variety of health measures. For example, bisphenol A biomarker data have been examined for associations with heart disease, obesity, type 2 diabetes, allergic asthma, metabolic syndrome, peripheral arterial disease, immune dysfunction, and markers of other chronic diseases (see Supplemental Material, Table S2). The vast array of potential associations between chemical biomarkers and health measures encourages research of this nature; indeed, multiple health measures (> 20, in some cases) have been examined for associations with biomarkers in almost all chemical groups (e.g., PAHs, PFCs, phthalates, pesticides, metals/metalloids) (see Supplemental Material, Table S2).

*Challenges and opportunities for health association studies*. Interpreting results for thousands of conceivable associations is a daunting task (Greenland 2008; Patel and Ioannidis 2014). Newer studies have therefore begun to simultaneously evaluate relationships between chemical biomarkers and health measures as part of environment-wide association studies (EWAS) (Patel et al. 2010, 2012, 2013, 2014). These studies better address statistical challenges related to multiple comparisons because more systematic methods are utilized. For NHANES-related health association studies to be considered in a risk assessment context, however, best practices are still needed for interpreting reported associations against the background of all possible associations (real and spurious). One approach is to compare a reported association with the median association among related biomarkers in a specific chemical category (Patel and Ioannidis 2014). This comparison indicates whether a reported association is remarkable relative to background, yet is dependent on predefined categories. An alternative approach is to first comprehensively test all possible associations and then report the strength of a single association relative to all results. Although this approach can be computationally prohibitive depending on model complexity, computationally efficient methods, such as frequent itemset mining, are now being systematically applied to NHANES data sets (Bell and Edwards 2014, 2015).

Specific attention has been given to the cross-sectional design of NHANES as it impacts studies of health association (LaKind et al. 2012). Notably, concurrent measures of biomarkers and health measures from NHANES are not useful for demonstrating temporality. Therefore, NHANES data alone are not well-suited for evaluating causation (or reverse causation) (Hill 1965), and health association studies often require follow-up targeted analysis. Furthermore, single spot measurements of chemical biomarkers in NHANES may not be reliable surrogates of average or peak exposure levels (Aylward et al. 2014; Bradman et al. 2013) and may not be relevant to exposures experienced during critical life stages. Studies have shown that large measurement error associated with spot measures (mostly reflecting exposure variability and rapid biological clearance) can contribute to exposure misclassification and increase the likelihood for biased statistical associations (Armstrong 1998; Jurek et al. 2006). Short-lived biomarkers in particular are prone to these challenges (Lin et al. 2005; Sobus et al. 2010b). Because some short-lived biomarkers are increasingly a focus of health association studies [e.g., environmental phenols and phthalates (Figure 4)], there is a need for methods that properly address measurement error. There is also a need for guidance on interpreting statistical associations between concurrent measures of short-lived biomarkers and chronic disease (LaKind et al. 2012).

Other challenges for health association studies stem not from the NHANES study design, but from the actual chemical biomarkers and health measures and from the methods used for their quantitation. Regarding health measures, the presence of illness among NHANES participants is often determined by self-report. In some cases, preclinical disease states and/or transient health events may not be appropriately captured, leading to altered associations between exposure and health. Rare illnesses also pose challenges for health association studies in that few cases can be linked to environmental exposures. Regarding chemical biomarkers, issues related to specificity, method sensitivity, and biological relevance are well documented and generally agreed upon (NRC 2006; Sobus et al. 2010a; Zelenka et al. 2011). Other issues, however, are still topics of intense debate. For example, a consensus has not been reached on how and when to adjust specific biomarkers for biological matrix effects. Levels of urinary and blood-based biomarkers, in particular, may require adjustment for variable urine output and lipid content, respectively. A recent study found that the direction (±), magnitude, and significance of associations between urinary phthalate metabolites and body size (waist circumference and body mass index) can differ depending on adjustments to the biomarkers (e.g., creatinine-adjusted vs. unadjusted concentration) (Christensen et al. 2014). These results highlight a clear need for standardized biomarker adjustment and analysis practices.

Guidance documents exist that can aid the planning, analysis, reporting, and interpretation of health association studies (Rooney et al. 2014; Vandenbroucke et al. 2007). In particular, the Biomonitoring, Environmental Epidemiology, and Short-Lived Chemicals (BEES-C) instrument developed by Lakind et al. (2014) targets critical issues that are unique to studies of short-lived chemical biomarkers. This instrument can be used for assessing the quality of health association research based on epidemiological study design and biomarker selection and measurement. It therefore serves as a resource for those planning studies using NHANES chemical biomarker data or those looking to evaluate published studies as part of a weight-of-evidence assessment. Discussions and evaluations surrounding the BEES-C instrument and other guidance documents are needed in order to clearly define and communicate best practices for health association studies involving NHANES data.

*Challenges and opportunities for exposure assessment studies*. Challenges exist for certain exposure assessment studies just as they do for health association studies. For example, measurement error can bias statistical associations between exposure metrics (e.g., dietary information, occupation) and chemical biomarker levels. This bias can impact the identification of important exposure sources and pathways for target chemicals. From a risk assessment standpoint, however, the most important challenges are those faced when linking chemical biomarker measurements to exposure levels. Because models are generally required to make these linkages, results are prone to error stemming from both the models themselves and the data inputs. Based on our review, studies that utilized models fell into two general categories: *a*) those that reconstructed exposure levels from NHANES biomarker data (reverse modeling), and *b*) those that compared biomarker measurements to model-predicted biomarker estimates (forward modeling). These forward- and reverse-modeling studies serve two key functions that support chemical risk assessment. The first is evaluation/calibration of exposure and/or pharmacokinetic models for improved exposure estimation. For example, in a recent forward-modeling study, Xue et al. (2010) compared NHANES urinary arsenic concentrations to levels predicted using the U.S. EPA’s Stochastic Human Exposure and Dose Simulation (SHEDS) model in order to quantify total exposure levels and determine major exposure contributors. In addition, in a recent reverse-modeling study, Wambaugh et al. (2013) reconstructed exposures from urinary concentrations of 82 NHANES chemicals and used these estimates to calibrate predictions from far-field mass balance human exposure models. The second key function of modeling studies is the comparison of biomarker levels to toxicological benchmarks such as no observed adverse effect levels (NOAELs) or RfDs. An example based on reverse modeling was given by Blount et al. (2007), who used urinary perchlorate data from NHANES to reconstruct total daily doses for comparison to the existing perchlorate RfD. Examples based on forward modeling are found in the growing body of literature related to biomonitoring equivalents (BE). Developed by Hays and colleagues, BEs are defined as levels of chemicals/metabolites in biological media that are consistent with exposure at a guidance level (Hays et al. 2007). These values are used to screen and prioritize chemicals based on the proximity of measured biomarker levels to estimated BEs. BEs and similar values were recently used to evaluate population exposures to > 100 chemicals monitored in NHANES, thus demonstrating the broad applicability of this approach (Aylward et al. 2013). Guidance documents exist for those looking to develop, apply, and interpret BEs with respect to NHANES biomarker data (Hays et al. 2008; LaKind et al. 2008).

Based on our review, models used for forward (biomarker) and reverse (exposure) predictions varied tremendously in terms of their complexity, ranging from simple analytical models to complex physiologically based pharmacokinetic (PBPK) models involving Markov Chain Monte Carlo analyses (Allen et al. 2007; Lyons et al. 2008). In any study, the ability to make accurate exposure or biomarker predictions is dependent upon the model applicability (e.g., how well the model describes the exposure–biomarker relationship), existing knowledge about the likely exposure scenarios (e.g., frequency of exposure), and measurement error (Tan et al. 2012). Challenges related to measurement error stem from a lack of repeated chemical biomarker measurements in NHANES. Spot biomarker distributions may not reflect distributions of average biomarker concentrations, which can only be obtained from repeated measures. For example, distribution tails (e.g., 5th and 95th percentiles) are often wider for spot measurements, particularly when examining short-lived biomarkers (Aylward et al. 2014; Christensen et al. 2012; Koch et al. 2014; Sobus et al. 2011). Thus, most NHANES-related studies have interpreted the median (or other central tendency estimate) of a spot biomarker distribution with respect to an exposure level of interest (Aylward et al. 2013; Wambaugh et al. 2013). Although this approach informs exposures to the U.S. population as a whole, it does not fully utilize the data in the upper percentiles, where there is increased probability of higher exposures. A recent article addressed this issue by offering a mathematical approach to estimate distributions of average biomarker levels given distributions of spot measurements (Pleil and Sobus 2013). This approach can be used to enhance exposure reconstruction models and calculate population exceedance against chronic exposure-based reference levels [i.e., the percentage of the U.S. population (or subset) with inferred average exposure in excess of a reference level].

A second issue related to modeling and the lack of repeated measurements in NHANES is the inability to interpret biomarker results for individual participants with respect to their chemical exposures. Especially for a short-lived biomarker, a single high measurement may reflect a consistently high exposure or a single recent elevated exposure. Such a determination generally cannot be made, given a lack of supplemental exposure data in NHANES. Therefore, individuals’ measurements are often collectively considered, based on measures of central tendency, to make population inferences. As an alternative, a stochastic modeling method was recently published that allows preliminary exposure evaluation at the individual participant level (Phillips et al. 2014). This method combines exposure models and PBPK models to predict biomarker distributions that are consistent with a reference exposure level. Measurements from NHANES individuals are then interpreted probabilistically with respect to the reference level. The goal of this approach is to improve assessments of population exposures by fully utilizing participant-level biomarker measurements, particularly those at the upper percentiles of measurement distributions.

*Need for best practices*. NHANES chemical biomarker data are primarily intended to provide references ranges for the U.S. population, track trends in chemical exposures, identify exposure disparities among population subsets, and set priorities for targeted research studies. The National Center for Health Statistics (NCHS) has developed extensive guidance materials that support the proper statistical analysis of these data (CDC 2010). These materials articulate important features of the NHANES design and correct procedures for data acquisition (e.g., downloading data sets, locating variables), management (e.g., merging and appending data files), and analysis. Key guidance relates to the appropriate use of sample weight and sample design variables, along with considerations for limits of detection, outlier observations, data transformations, matrix adjustments, and laboratory procedure changes. The reproducibility of NHANES-based research requires adherence to these well-documented practices. Yet, as procedures for data analysis grow and evolve, so must guidance and recommendations. This article has highlighted cutting-edge approaches for interpreting NHANES chemical biomarker data in a manner that can further aid risk assessment/management activities. Methods now exist to systematically evaluate data across chemicals, health measures, and study participants. These methods are facilitating multichemical/group assessments that will help prioritize needs for follow-up targeted assessments. Moving forward, it will become increasingly important to define, communicate, and follow best practices for assessing biomarkers of individual chemicals, chemical groups, and the expanding NHANES chemical inventory. Table 1 gives a summary of our recommendations for meeting these goals, as well as supporting references that provide methods and/or additional discussion related to our recommendations. It will also be valuable to discuss best practices with other countries (e.g., Canada, Germany, Korea) that measure chemical biomarkers in representative samples of the national population. These steps will ensure that results of screening-level evaluations, prioritizations, and targeted analyses are scientifically defensible and used appropriately in decision making.

**Table 1:** Recommendations for developers and users of NHANES chemical biomarker data.

Recommendations	Supporting references
Follow NCHS recommendations when acquiring, managing, and analyzing NHANES biomarker data	CDC 2010
Utilize guidance documents when planning, executing, communicating, and reviewing research based on NHANES biomarker data	Hays et al. 2008; LaKind et al. 2008, 2014
Establish best practices for addressing measurement variability/error in both exposure assessment and health association studies	LaKind et al. 2014; Phillips et al. 2014; Pleil and Sobus 2013
Establish best practices for adjusting biomarker measurements for biological matrix effects in both exposure assessment and health association studies	Christensen et al. 2014; LaKind et al. 2014
Establish best practices for using NHANES biomarker data to evaluate/calibrate exposure models and/or pharmacokinetic models	Wambaugh et al. 2013; Xue et al. 2010
Perform systematic evaluations of relationships between stressors (chemical and nonchemical) and effects of stressors on human health	Bell and Edwards 2014; Patel et al. 2013
Establish best practices for interpreting individual exposure–health associations against background of all possible associations	Bell and Edwards 2015; Patel and Ioannidis 2014
Establish best practices for interpreting associations between concurrent measures of short-lived biomarkers and health status	LaKind et al. 2012, 2014
Supplement existing (targeted) NHANES biomarker panels using nontargeted analyses of biological samples	Rappaport 2012; Rappaport et al. 2014
Abbreviations: NCHS, National Center for Health Statistics; NHANES, National Health and Nutrition Examination Survey.	

*Limitations of this study*. A major goal of this study was to evaluate trends in the uses of NHANES chemical biomarker data using a sample of publications. There are some limitations with the methods used for sample selection and analysis. First, all publications evaluated here were identified using the PubMed Advanced Search Builder. Articles not indexed in PubMed were not captured in our search. Second, our search was restricted to publications that explicitly listed NHANES in the title/abstract. It is likely that there are some studies that utilized NHANES data without mentioning the survey name in the publication title/abstract. Third, all PubMed searches included query terms related to “U.S.” in the title/abstract; this search criterion guarded against the inclusion of non-U.S. NHANES studies. Results from preliminary analyses (see Supplemental Material, Table S1) showed that about 42% of the yearly NHANES-related publications were omitted from our search after including the “U.S.” query terms. This ultimately restricted the number of publications that were curated and included in the trends analysis. Fourth, electronic publications (epubs) for 2013 were included in the results from all PubMed searches and manual curations. The inclusion of these results elevated the number of 2013 publications for each search step. However, only 2 of 44 publications in 2013 were included as part of the final manually curated list, indicating that this should have little or no impact on the trends seen.

The final limitation of this study relates to the binning of publications (second curation) based on chemical group. NHANES biomarkers have been defined with slight differences across survey years. Therefore, groupings we used were based on both recent NHANES documents and empirical evidence from the selected literature (specifically, biomarkers that have been routinely coexamined were grouped together). Using this approach, the number of biomarkers across chemical groups was variable. For example, the group “environmental phenols” included few biomarkers, whereas “pesticides” included many biomarkers from a variety of classes. No attempt was made to weigh groups based on the number of biomarkers. This has implications when designating certain publications as “multi-group.” Specifically, some multi-group publications examined many biomarkers across all chemical groups. Others investigated few biomarkers across only two groups. A few publications examined many biomarkers as part of one large chemical group and were not considered “multi-group.”

Each of the limitations discussed above may have introduced some amount of error or bias into our analysis. The main objective of this investigation, however, was to gain a better understanding of the primary uses of NHANES data based on a sample of studies from the published literature. The trends observed here do indeed highlight existing research challenges and opportunities to advance the science. Future investigations of NHANES data usage will provide further information regarding the recent trends observed in the present study.

## Conclusions

This article is among the first to investigate trends in the uses of NHANES chemical biomarker data. Extrapolating from our results, it is expected that > 100 articles will be published each year that examine these data. Given this usage, it is likely that NHANES data will impact chemical risk assessment decisions. New methods and guidelines are rapidly emerging to address challenges that face analysis, reporting, and interpretation of the NHANES data. Because exposure assessment and health association studies are moving toward multichemical/group assessments (Belova et al. 2013; Patel et al. 2013; Wambaugh et al. 2013), it is increasingly important to define and adopt best research practices. Such measures will allow the full potential of the NHANES to be realized and defensible decisions based on the data and emerging science to be made.

## Supplemental Material

(523 KB) PDF
