# Supplementary material for: Uses of NHANES Biomarker Data for Chemical Risk Assessment: Trends, Challenges, and Opportunities
Source: Environ Health Perspect. 2015 Apr 10;123(10):919–27. doi: 10.1289/ehp.1409177 (PMC4590763; doi:10.1289/ehp.1409177)
Supplement: (523 KB) PDF [file ehp.1409177.s001.acco.pdf]

Environ Health Perspect
doi: 10.1289/ehp.1409177

Note to Readers: *EHP* strives to ensure that all journal content is accessible to all readers. However, some figures and Supplemental Material published in *EHP* articles may not conform to 508 standards due to the complexity of the information being presented. If you need assistance accessing journal content, please contact ehp508@niehs.nih.gov. Our staff will work with you to assess and meet your accessibility needs within 3 working days.

Supplemental Material

Uses of NHANES Biomarker Data for Chemical Risk Assessment: Trends, Challenges, and Opportunities

Jon R. Sobus, Robert S. DeWoskin, Yu-Mei Tan, Joachim D. Pleil, Martin Blake Phillips, Barbara Jane George, Krista Christensen, Dina M. Schreinemachers, Marc A. Williams, Elaine A. Cohen Hubal, and Stephen W. Edwards

Table of Contents

Table S1. Search focus, PubMed search strings, and number of publications associated with each step of the literature review. (PubMed searches were performed in May 2014.)

Table S2. Publications that utilized chemical biomarker data from the U.S. NHANES. Papers are organized by chemical group, analysis category, then year of publication.

References

Table S1. Search focus, PubMed search strings, and number of publications associated with each step of the literature review. (PubMed searches were performed in May 2014.)

Search step	Search focus	Search string (PubMed advanced search builder)	# of publications
Preliminary search[a]	NHANES	(((NHANES[Title/Abstract]) OR (National Health and Nutrition Examination Survey[Title/Abstract])) AND ("1999/01/01"[Date - Publication] : "2013/12/31"[Date - Publication]))	5606
Step 1	NHANES + U.S.	(((NHANES[Title/Abstract]) OR (National Health and Nutrition Examination Survey[Title/Abstract])) AND ("1999/01/01"[Date - Publication] : "2013/12/31"[Date - Publication]) AND ((United States[Title/Abstract]) OR (U.S.A.[Title/Abstract]) OR (USA[Title/Abstract]) OR (U.S.[Title/Abstract]) OR (US[Title/Abstract])))	3224
Step 2	NHANES + U.S. + biomarkers	(((NHANES[Title/Abstract]) OR (National Health and Nutrition Examination Survey[Title/Abstract])) AND ("1999/01/01"[Date - Publication] : "2013/12/31"[Date - Publication]) AND ((United States[Title/Abstract]) OR (U.S.A.[Title/Abstract]) OR (USA[Title/Abstract]) OR (U.S.[Title/Abstract]) OR (US[Title/Abstract])) AND ((biomarker[Title/Abstract]) OR (biomarkers[Title/Abstract]) OR (biomonitoring[Title/Abstract]) OR (urine[Title/Abstract]) OR (urinary[Title/Abstract]) OR (blood[Title/Abstract]) OR (serum[Title/Abstract])))	1382
Manual curation	NHANES + U.S. + chemical biomarkers	NA[b]	273

[a]Results from the preliminary search were not included in the analysis since non-U.S. NHANES studies were identified. [b]Manual curation involved a review of titles and abstracts for publications identified in step 2 of the review.

Table S2. Publications that utilized chemical biomarker data from the U.S. NHANES. Papers are organized by chemical group, analysis category, then year of publication.

Publication title	Chemical group	Analysis category	Reference
Elevated house dust and serum concentrations of PBDEs in California: unintended consequences of furniture flammability standards?	BFRs	exposure assessment	(Zota et al. 2008)
Serum concentrations of polybrominated diphenyl ethers (PBDEs) and polybrominated biphenyl (PBB) in the United States population: 2003-2004.	BFRs	exposure assessment	(Sjodin et al. 2008)
Serum levels of polybrominated diphenyl ethers (PBDEs) in foam recyclers and carpet installers working in the United States.	BFRs	exposure assessment	(Stapleton et al. 2008)
Diet contributes significantly to the body burden of PBDEs in the general U.S. population.	BFRs	exposure assessment	(Fraser et al. 2009)
Improving infant exposure and health risk estimates: using serum data to predict polybrominated diphenyl ether concentrations in breast milk.	BFRs	exposure assessment	(Marchitti et al. 2013)
Association of brominated flame retardants with diabetes and metabolic syndrome in the U.S. population, 2003-2004.	BFRs	health association	(Lim et al. 2008)
Age-specific reference ranges for polychlorinated biphenyls (PCB) based on the NHANES 2001-2002 survey.	dioxins/furans/PCBs	exposure assessment	(Nichols et al. 2007)

**Publication title**	**Chemical group**	**Analysis category**	**Reference**
Evaluation of PCDD/F and dioxin-like PCB serum concentration data from the 2001-2002 National Health and Nutrition Examination Survey of the United States population.	dioxins/furans/PCBs	exposure assessment	(Ferriby et al. 2007)
Addendum to: Evaluation of PCDD/F and dioxin-like PCB serum concentration data from the 2001-2002 National Health and Nutrition Examination Survey of the United States population.	dioxins/furans/PCBs	exposure assessment	(Scott et al. 2008)
Serum dioxin levels in residents of Calcasieu and Lafayette parishes, Louisiana with comparison to the US population.	dioxins/furans/PCBs	exposure assessment	(Wong le et al. 2008)
Evaluation of background exposures of Americans to dioxin-like compounds in the 1990s and the 2000s.	dioxins/furans/PCBs	exposure assessment	(Lorber et al. 2009)
Perspective on serum dioxin levels in the United States: an evaluation of the NHANES data.	dioxins/furans/PCBs	exposure assessment	(LaKind et al. 2009)
Regression models to estimate total polychlorinated biphenyls in the general US population: 2001-2002 and 2003-2004.	dioxins/furans/PCBs	exposure assessment	(Jain and Wang 2010)
Association of caffeine consumption and smoking status with the serum concentrations of polychlorinated biphenyls, dioxins, and furans in the general U.S. population: NHANES 2003-2004.	dioxins/furans/PCBs	exposure assessment	(Jain and Wang 2011)
Use of pooled samples from the National Health and Nutrition Examination Survey.	dioxins/furans/PCBs	exposure assessment	(Caudill 2012)

Publication title	Chemical group	Analysis category	Reference
Estimation of age- and sex-specific background human serum concentrations of PCDDs, PCDFs, and PCBs in the UMDES and NHANES populations.	dioxins/furans/PCBs	exposure assessment	(Q Chen et al. 2013)
Bisphenol A (BPA) daily intakes in the United States: estimates from the 2003-2004 NHANES urinary BPA data.	environmental phenols	exposure assessment	(Lakind and Naiman 2008)
Concentrations of the sunscreen agent benzophenone-3 in residents of the United States: National Health and Nutrition Examination Survey 2003-2004.	environmental phenols	exposure assessment	(Calafat et al. 2008a)
Exposure of the U.S. population to bisphenol A and 4-tertiary-octylphenol: 2003-2004.	environmental phenols	exposure assessment	(Calafat et al. 2008c)
Urinary concentrations of triclosan in the U.S. population: 2003-2004.	environmental phenols	exposure assessment	(Calafat et al. 2008b)
Urinary concentrations of four parabens in the U.S. population: NHANES 2005-2006.	environmental phenols	exposure assessment	(Calafat et al. 2010)
Daily intake of bisphenol A and potential sources of exposure: 2005-2006 National Health and Nutrition Examination Survey.	environmental phenols	exposure assessment	(Lakind and Naiman 2011)
Comparing United States and Canadian population exposures from National Biomonitoring Surveys: bisphenol A intake as a case study.	environmental phenols	exposure assessment	(LaKind et al. 2012)
Urinary bisphenol A concentrations in girls from rural and urban Egypt: a pilot study.	environmental phenols	exposure assessment	(Nahar et al. 2012)

Publication title	Chemical group	Analysis category	Reference
Association of urinary bisphenol A concentration with medical disorders and laboratory abnormalities in adults.	environmental phenols	health association	(Lang et al. 2008)
Association of urinary bisphenol A concentration with heart disease: evidence from NHANES 2003/06.	environmental phenols	health association	(Melzer et al. 2010b)
The impact of bisphenol A and triclosan on immune parameters in the U.S. population, NHANES 2003-2006.	environmental phenols	health association	(Clayton et al. 2011)
Urinary bisphenol A and obesity: NHANES 2003-2006.	environmental phenols	health association	(Carwile and Michels 2011)
Urinary bisphenol A and type-2 diabetes in U.S. adults: data from NHANES 2003-2008.	environmental phenols	health association	(Silver et al. 2011)
Association of urinary bisphenol A concentration with allergic asthma: results from the National Health and Nutrition Examination Survey 2005-2006.	environmental phenols	health association	(Vaidya and Kulkarni 2012)
Bisphenol A and Metabolic Syndrome: Results from NHANES.	environmental phenols	health association	(Teppala et al. 2012)
Bisphenol A and peripheral arterial disease: results from the NHANES.	environmental phenols	health association	(Shankar et al. 2012)
Bisphenol A and chronic disease risk factors in US children.	environmental phenols	health association	(Eng et al. 2013)
Bisphenol A exposure is associated with low-grade urinary albumin excretion in children of the United States.	environmental phenols	health association	(Trasande et al. 2013b)

Publication title	Chemical group	Analysis category	Reference
Relationship between urinary triclosan and paraben concentrations and serum thyroid measures in NHANES 2007-2008.	environmental phenols	health association	(Koeppe et al. 2013)
Urinary bisphenol A and obesity in U.S. children.	environmental phenols	health association	(Bhandari et al. 2013)
Relationship of ascorbic acid to blood lead levels.	metals/metalloids	exposure assessment	(Simon and Hudes 1999)
Blood lead levels in young children-United States and selected states, 1996-1999.	metals/metalloids	exposure assessment	(CDC 2000)
Elevated blood lead levels and blood lead screening among US children aged one to five years: 1988-1994.	metals/metalloids	exposure assessment	(Kaufmann et al. 2000)
Exposure of the U.S. population aged 6 years and older to cadmium: 1988-1994.	metals/metalloids	exposure assessment	(Paschal et al. 2000)
Blood and hair mercury levels in young children and women of childbearing age-United States, 1999.	metals/metalloids	exposure assessment	(CDC 2001)
Urinary cadmium elimination as a biomarker of exposure for evaluating a cadmium dietary exposure-biokinetics model.	metals/metalloids	exposure assessment	(Choudhury et al. 2001)
An exposure assessment for methylmercury from seafood for consumers in the United States.	metals/metalloids	exposure assessment	(Carrington and Bolger 2002)
Economic gains resulting from the reduction in children's exposure to lead in the United States.	metals/metalloids	exposure assessment	(Grosse et al. 2002)

Publication title	Chemical group	Analysis category	Reference
Lead-based paint health risk assessment in dependent children living in military housing.	metals/metalloids	exposure assessment	(Stroop et al. 2002)
The relationship between blood lead levels and periodontal bone loss in the United States, 1988-1994.	metals/metalloids	exposure assessment	(Dye et al. 2002)
Blood mercury levels in US children and women of childbearing age, 1999-2000.	metals/metalloids	exposure assessment	(Schober et al. 2003)
Changing trends in the epidemiology of pediatric lead exposure: interrelationship of blood lead and ZPP concentrations and a comparison to the US population.	metals/metalloids	exposure assessment	(Soldin et al. 2003)
Prevalence of blood lead levels >or= 5 micro g/dL among US children 1 to 5 years of age and socioeconomic and demographic factors associated with blood of lead levels 5 to 10 micro g/dL, Third National Health and Nutrition Examination Survey, 1988-1994.	metals/metalloids	exposure assessment	(Bernard and McGeehin 2003)
Second-hand smoke exposure and blood lead levels in U.S. children.	metals/metalloids	exposure assessment	(Mannino et al. 2003)
Surveillance for elevated blood lead levels among children--United States, 1997-2001.	metals/metalloids	exposure assessment	(Meyer et al. 2003)
An intervention analysis for the reduction of exposure to methylmercury from the consumption of seafood by women of child-bearing age.	metals/metalloids	exposure assessment	(Carrington et al. 2004)

Publication title	Chemical group	Analysis category	Reference
Blood lead levels in U.S. workers, 1988-1994.	metals/metalloids	exposure assessment	(Yassin et al. 2004)
Blood mercury levels in young children and childbearing-aged women-United States, 1999-2002.	metals/metalloids	exposure assessment	(CDC 2004)
Blood organic mercury and dietary mercury intake: National Health and Nutrition Examination Survey, 1999 and 2000.	metals/metalloids	exposure assessment	(Mahaffey et al. 2004)
Bone density-related predictors of blood lead level among peri- and postmenopausal women in the United States: The Third National Health and Nutrition Examination Survey, 1988-1994.	metals/metalloids	exposure assessment	(Nash et al. 2004)
Urinary cadmium levels in the US working population, 1988-1994.	metals/metalloids	exposure assessment	(Yassin and Martonik 2004)
Active and passive smoking and blood lead levels in U.S. adults: data from the Third National Health and Nutrition Examination Survey.	metals/metalloids	exposure assessment	(Mannino et al. 2005)
Blood lead levels-United States, 1999-2002.	metals/metalloids	exposure assessment	(CDC 2005)
Demographic and socioeconomic factors associated with blood lead levels among Mexican-American children and adolescents in the United States.	metals/metalloids	exposure assessment	(Morales et al. 2005)
Determinants of the blood lead level of US women of reproductive age.	metals/metalloids	exposure assessment	(Lee et al. 2005)

Publication title	Chemical group	Analysis category	Reference
Mercury exposure: medical and public health issues.	metals/metalloids	exposure assessment	(Mahaffey 2005)
Urinary mercury concentrations associated with dental restorations in adult women aged 16-49 years: United States, 1999-2000.	metals/metalloids	exposure assessment	(Dye et al. 2005)
Blood lead concentrations in children and method of water fluoridation in the United States, 1988-1994.	metals/metalloids	exposure assessment	(Macek et al. 2006)
Blood lead levels in primary school children in Trinidad and Tobago.	metals/metalloids	exposure assessment	(Rajkumar et al. 2006)
Validation of a 20-year forecast of US childhood lead poisoning: Updated prospects for 2010.	metals/metalloids	exposure assessment	(Jacobs and Nevin 2006)
Use of Markov Chain Monte Carlo analysis with a physiologically-based pharmacokinetic model of methylmercury to estimate exposures in US women of childbearing age.	metals/metalloids	exposure assessment	(Allen et al. 2007)
Estimated burden of blood lead levels 5 microg/dl in 1999-2002 and declines from 1988 to 1994.	metals/metalloids	exposure assessment	(Iqbal et al. 2008)
Methylmercury and omega-3 fatty acids: co-occurrence of dietary sources with emphasis on fish and shellfish.	metals/metalloids	exposure assessment	(Mahaffey et al. 2008)
Adult women's blood mercury concentrations vary regionally in the United States: association with patterns of fish consumption (NHANES 1999-2004).	metals/metalloids	exposure assessment	(Mahaffey et al. 2009)

**Publication title**	**Chemical group**	**Analysis category**	**Reference**
Exposure of U.S. children to residential dust lead, 1999-2004: II. The contribution of lead-contaminated dust to children's blood lead levels.	metals/metalloids	exposure assessment	(Dixon et al. 2009)
Herbal supplement use and blood lead levels of United States adults.	metals/metalloids	exposure assessment	(Buettner et al. 2009)
Levels of urinary total and speciated arsenic in the US population: National Health and Nutrition Examination Survey 2003-2004.	metals/metalloids	exposure assessment	(Caldwell et al. 2009a)
Tobacco smoke exposure and levels of urinary metals in the U.S. youth and adult population: the National Health and Nutrition Examination Survey (NHANES) 1999-2004.	metals/metalloids	exposure assessment	(Richter et al. 2009)
Total blood mercury concentrations in the U.S. population: 1999-2006.	metals/metalloids	exposure assessment	(Caldwell et al. 2009b)
Trends in blood lead levels and blood lead testing among US children aged 1 to 5 years, 1988-2004.	metals/metalloids	exposure assessment	(Jones et al. 2009)
Biological variation of laboratory analytes based on the 1999-2002 National Health and Nutrition Examination Survey.	metals/metalloids	exposure assessment	(Lacher et al. 2010)
Blood cadmium levels in women of childbearing age vary by race/ethnicity.	metals/metalloids	exposure assessment	(Mijal and Holzman 2010)
Blood lead and mercury levels in pregnant women in the United States, 2003-2008.	metals/metalloids	exposure assessment	(Jones et al. 2010)

Publication title	Chemical group	Analysis category	Reference
Heavy metals exposures among Mexican farmworkers in eastern North Carolina.	metals/metalloids	exposure assessment	(Quandt et al. 2010)
Physiologically based pharmacokinetic (PBPK) tool kit for environmental pollutants--metals.	metals/metalloids	exposure assessment	(Ruiz et al. 2010)
Probabilistic Modeling of Dietary Arsenic Exposure and Dose and Evaluation with 2003-2004 NHANES Data.	metals/metalloids	exposure assessment	(Xue et al. 2010)
Geographic region of residence and blood lead levels in US children: results of the National Health and Nutrition Examination Survey.	metals/metalloids	exposure assessment	(Scott and Nguyen 2011)
Seafood intake and urine concentrations of total arsenic, dimethylarsinate and arsenobetaine in the US population.	metals/metalloids	exposure assessment	(Navas-Acien et al. 2011)
Smoking status and urine cadmium above levels associated with subclinical renal effects in U.S. adults without chronic kidney disease.	metals/metalloids	exposure assessment	(Mortensen et al. 2011)
Blood lead levels in the United States “oldest-old" population.	metals/metalloids	exposure assessment	(Vearrier and Greenberg 2012)
Reduction in cadmium exposure in the United States population, 1988-2008: the contribution of declining smoking rates.	metals/metalloids	exposure assessment	(Tellez-Plaza et al. 2012a)
Rice consumption and urinary arsenic concentrations in U.S. children.	metals/metalloids	exposure assessment	(Davis et al. 2012)

**Publication title**	**Chemical group**	**Analysis category**	**Reference**
Secondhand tobacco smoke: a source of lead exposure in US children and adolescents.	metals/metalloids	exposure assessment	(Apostolou et al. 2012)
A multi-level model of blood lead as a function of air lead.	metals/metalloids	exposure assessment	(Richmond-Bryant et al. 2013)
Blood lead levels in children aged 1-5 years - United States, 1999-2010.	metals/metalloids	exposure assessment	(Wheeler and Brown 2013)
Calibrating sensitivity analyses to observed covariates in observational studies.	metals/metalloids	exposure assessment	(Hsu and Small 2013)
Rice consumption and urinary concentrations of arsenic in US adults.	metals/metalloids	exposure assessment	(Wei et al. 2014)
Trends in tobacco smoke exposure and blood lead levels among youths and adults in the United States: the National Health and Nutrition Examination Survey, 1999-2008.	metals/metalloids	exposure assessment	(Richter et al. 2013)
Urinary cadmium in the 1999-2008 U.S. National Health and Nutrition Examination Survey (NHANES).	metals/metalloids	exposure assessment	(Riederer et al. 2013)
Association of dental caries and blood lead levels.	metals/metalloids	health association	(Moss et al. 1999)
Blood lead concentration and children's anthropometric dimensions in the Third National Health and Nutrition Examination Survey (NHANES III), 1988-1994.	metals/metalloids	health association	(Ballew et al. 1999)

**Publication title**	**Chemical group**	**Analysis category**	**Reference**
Serum magnesium and ischaemic heart disease: findings from a national sample of US adults.	metals/metalloids	health association	(Ford 1999)
Cognitive deficits associated with blood lead concentrations <10 microg/dL in US children and adolescents.	metals/metalloids	health association	(Lanphear et al. 2000)
Serum copper concentration and coronary heart disease among US adults.	metals/metalloids	health association	(Ford 2000)
Blood lead levels and mortality.	metals/metalloids	health association	(Lustberg and Silbergeld 2002)
The association of blood lead level and cancer mortality among whites in the United States.	metals/metalloids	health association	(Jemal et al. 2002)
Blood lead and chronic kidney disease in the general United States population: results from NHANES III.	metals/metalloids	health association	(Muntner et al. 2003)
Blood lead levels and sexual maturation in U.S. girls: the Third National Health and Nutrition Examination Survey, 1988-1994.	metals/metalloids	health association	(Wu et al. 2003)
Blood lead, blood pressure, and hypertension in perimenopausal and postmenopausal women.	metals/metalloids	health association	(Nash et al. 2003)
Can the National Health and Nutrition Examination Survey III (NHANES III) data help resolve the controversy over low blood lead levels and neuropsychological development in children?	metals/metalloids	health association	(Stone and Reynolds 2003)

**Publication title**	**Chemical group**	**Analysis category**	**Reference**
Urinary cadmium, impaired fasting glucose, and diabetes in the NHANES III.	metals/metalloids	health association	(Schwartz et al. 2003)
Lead, cadmium, smoking, and increased risk of peripheral arterial disease.	metals/metalloids	health association	(Navas-Acien et al. 2004)
Serum iron, copper and zinc concentrations and risk of cancer mortality in US adults.	metals/metalloids	health association	(Wu et al. 2004)
Urinary cadmium levels predict lower lung function in current and former smokers: data from the Third National Health and Nutrition Examination Survey.	metals/metalloids	health association	(Mannino et al. 2004)
Blood mercury level and blood pressure among US women: results from the National Health and Nutrition Examination Survey 1999-2000.	metals/metalloids	health association	(Vupputuri et al. 2005)
Continued decline in blood lead levels among adults in the United States: the National Health and Nutrition Examination Surveys.	metals/metalloids	health association	(Muntner et al. 2005)
Metals in urine and peripheral arterial disease.	metals/metalloids	health association	(Navas-Acien et al. 2005)
Blood lead below 0.48 micromol/L (10 microg/dL) and mortality among US adults.	metals/metalloids	health association	(Menke et al. 2006)
Blood lead levels and death from all causes, cardiovascular disease, and cancer: results from the NHANES III mortality study.	metals/metalloids	health association	(Schober et al. 2006)

Publication title	Chemical group	Analysis category	Reference
Exposures to environmental toxicants and attention deficit hyperactivity disorder in U.S. children.	metals/metalloids	health association	(Braun et al. 2006)
Graded associations of blood lead and urinary cadmium concentrations with oxidative-stress-related markers in the U.S. population: results from the Third National Health and Nutrition Examination Survey.	metals/metalloids	health association	(Lee et al. 2006)
Lead exposure and periodontitis in US adults.	metals/metalloids	health association	(Saraiva et al. 2007)
Arsenic exposure and prevalence of type 2 diabetes in US adults.	metals/metalloids	health association	(Navas-Acien et al. 2008)
Association of environmental toxicants and conduct disorder in U.S. children: NHANES 2001-2004.	metals/metalloids	health association	(Braun et al. 2008)
Cadmium exposure and hypertension in the 1999-2004 National Health and Nutrition Examination Survey (NHANES).	metals/metalloids	health association	(Tellez-Plaza et al. 2008)
The association of urinary cadmium with sex steroid hormone concentrations in a general population sample of US adult men.	metals/metalloids	health association	(Menke et al. 2008)
Urinary cadmium and osteoporosis in U.S. Women >or= 50 years of age: NHANES 1988-1994 and 1999-2004.	metals/metalloids	health association	(Gallagher et al. 2008)
Assessment of chronic mercury exposure within the U.S. population, National Health and Nutrition Examination Survey, 1999-2006.	metals/metalloids	health association	(Laks 2009)

Publication title	Chemical group	Analysis category	Reference
Association of environmental cadmium exposure with periodontal disease in U.S. adults.	metals/metalloids	health association	(Arora et al. 2009)
Association of tobacco and lead exposures with attention-deficit/hyperactivity disorder.	metals/metalloids	health association	(Froehlich et al. 2009)
Blood cadmium and lead and chronic kidney disease in US adults: a joint analysis.	metals/metalloids	health association	(Navas-Acien et al. 2009)
Blood lead levels and major depressive disorder, panic disorder, and generalized anxiety disorder in US young adults.	metals/metalloids	health association	(Bouchard et al. 2009)
Cadmium levels in urine and mortality among U.S. adults.	metals/metalloids	health association	(Menke et al. 2009)
Low-level population exposure to inorganic arsenic in the United States and diabetes mellitus: a reanalysis.	metals/metalloids	health association	(Steinmaus et al. 2009)
A population-based study of blood lead levels in relation to depression in the United States.	metals/metalloids	health association	(Golub et al. 2010)
Association between lead and cadmium and reproductive hormones in peripubertal U.S. girls.	metals/metalloids	health association	(Gollenberg et al. 2010)
Blood lead level and kidney function in US adolescents: The Third National Health and Nutrition Examination Survey.	metals/metalloids	health association	(Fadrowski et al. 2010)
Cadmium and peripheral arterial disease: gender differences in the 1999-2004 US National Health and Nutrition Examination Survey.	metals/metalloids	health association	(Tellez-Plaza et al. 2010)

**Publication title**	**Chemical group**	**Analysis category**	**Reference**
Cadmium, follicle-stimulating hormone, and effects on bone in women age 42-60 years, NHANES III.	metals/metalloids	health association	(Gallagher et al. 2010b)
Environmental cadmium and breast cancer risk.	metals/metalloids	health association	(Gallagher et al. 2010a)
Low level exposure to cadmium increases the risk of chronic kidney disease: analysis of the NHANES 1999-2006.	metals/metalloids	health association	(Ferraro et al. 2010)
Modification by ALAD of the association between blood lead and blood pressure in the U.S. population: results from the Third National Health and Nutrition Examination Survey.	metals/metalloids	health association	(Scinicariello 2010)
Urinary cadmium, osteopenia, and osteoporosis in the US population.	metals/metalloids	health association	(Wu et al. 2010)
Association of low-level blood lead and blood pressure in NHANES 1999-2006.	metals/metalloids	health association	(Scinicariello et al. 2011)
Blood lead levels in relation to cognitive function in older U.S. adults.	metals/metalloids	health association	(van Wijngaarden et al. 2011)
Heavy metals exposure and hearing loss in US adolescents.	metals/metalloids	health association	(Shargorodsky et al. 2011)
Total blood mercury and serum measles antibodies in US children, NHANES 2003-2004.	metals/metalloids	health association	(Gallagher et al. 2011)

Publication title	Chemical group	Analysis category	Reference
Total blood mercury, plasma homocysteine, methylmalonic acid and folate in US children aged 3-5 years, NHANES 1999-2004.	metals/metalloids	health association	(Gallagher and Meliker 2011)
Urine arsenic and hypertension in US adults: the 2003-2008 National Health and Nutrition Examination Survey.	metals/metalloids	health association	(Jones et al. 2011)
Urine arsenic concentration and obstructive pulmonary disease in the U.S. population.	metals/metalloids	health association	(Amster et al. 2011)
Association of homocysteine levels with blood lead levels and micronutrients in the US general population.	metals/metalloids	health association	(Lee et al. 2012)
Association of markers of chronic viral hepatitis and blood mercury levels in US reproductive-age women from NHANES 2001-2008: a cross-sectional study.	metals/metalloids	health association	(Sheehan et al. 2012)
Cadmium exposure and all-cause and cardiovascular mortality in the U.S. general population.	metals/metalloids	health association	(Tellez-Plaza et al. 2012b)
Cadmium exposure and cancer mortality in the Third National Health and Nutrition Examination Survey cohort.	metals/metalloids	health association	(Adams et al. 2012)
Cadmium exposure and neurodevelopmental outcomes in U.S. children.	metals/metalloids	health association	(Ciesielski et al. 2012)
Environmental cadmium and lead exposures and hearing loss in U.S. adults: the National Health and Nutrition Examination Survey, 1999 to 2004.	metals/metalloids	health association	(Choi et al. 2012)

Publication title	Chemical group	Analysis category	Reference
Lead and cadmium levels and balance and vestibular dysfunction among adult participants in the National Health and Nutrition Examination Survey (NHANES) 1999-2004.	metals/metalloids	health association	(KB Min et al. 2012)
Mercury and thyroid autoantibodies in U.S. women, NHANES 2007-2008.	metals/metalloids	health association	(Gallagher and Meliker 2012)
Temporal trend of cadmium exposure in the United States population suggests gender specificities.	metals/metalloids	health association	(Ferraro et al. 2012)
The association between urinary cadmium and frontal T wave axis deviation in the US adults.	metals/metalloids	health association	(Faramawi et al. 2012)
Urinary heavy metals and associated medical conditions in the US adult population.	metals/metalloids	health association	(Mendy et al. 2012)
Association between blood lead and walking speed in the National Health and Nutrition Examination Survey (NHANES 1999-2002).	metals/metalloids	health association	(Ji et al. 2013)
Association between total blood mercury and exhaled nitric oxide in US adults.	metals/metalloids	health association	(Min and Min 2013)
Associations between cadmium exposure and neurocognitive test scores in a cross-sectional study of US adults.	metals/metalloids	health association	(Ciesielski et al. 2013)
Associations of blood and urinary mercury with hypertension in U.S. adults: the NHANES 2003-2006.	metals/metalloids	health association	(Park et al. 2013)

Publication title	Chemical group	Analysis category	Reference
Blood cadmium is elevated in iron deficient U.S. children: a cross-sectional study.	metals/metalloids	health association	(Silver et al. 2013)
Cadmium exposure and liver disease among US adults.	metals/metalloids	health association	(Hyder et al. 2013)
Menopause and lead body burden among US women aged 45-55, NHANES 1999-2010.	metals/metalloids	health association	(Mendola et al. 2013)
Metals in blood and urine, and thyroid function among adults in the United States 2007-2008.	metals/metalloids	health association	(Christensen 2013)
Methylmercury and elemental mercury differentially associate with blood pressure among dental professionals.	metals/metalloids	health association	(Goodrich et al. 2013)
Potential health impacts of heavy metals on HIV-infected population in USA.	metals/metalloids	health association	(XH Xu et al. 2013)
Serum heavy metals and obstructive lung disease: results from the National Health and Nutrition Examination Survey.	metals/metalloids	health association	(Rokadia and Agarwal 2013)
Thyroid hormones in relation to lead, mercury, and cadmium exposure in the National Health and Nutrition Examination Survey, 2007-2008.	metals/metalloids	health association	(AM Chen et al. 2013)
Total blood mercury and rubella antibody concentrations in US children aged 6-11 years, NHANES 2003-2004.	metals/metalloids	health association	(Gallagher et al. 2013b)

Publication title	Chemical group	Analysis category	Reference
Total blood mercury levels and depression among adults in the United States: National Health and Nutrition Examination Survey 2005-2008.	metals/metalloids	health association	(Ng et al. 2013)
Levels of metabolites of organophosphate pesticides, phthalates, and bisphenol A in pooled urine specimens from pregnant women participating in the Norwegian Mother and Child Cohort Study (MoBa).	multi-group	exposure assessment	(Ye et al. 2009)
Serum concentrations of selected persistent organic pollutants in a sample of pregnant females and changes in their concentrations during gestation.	multi-group	exposure assessment	(Wang et al. 2009)
The CDC fourth national report on human exposure to environmental chemicals: what it tells us about our toxic burden and how it assist environmental medicine physicians.	multi-group	exposure assessment	(Crinnion 2010)
Blood and urine levels of long half-life toxicants by nativity among immigrants to the United States.	multi-group	exposure assessment	(Muennig et al. 2011)
Differential exposure biomarker levels among cigarette smokers and smokeless tobacco consumers in the National Health and Nutrition Examination Survey 1999-2008.	multi-group	exposure assessment	(Naufal et al. 2011)
Environmental chemicals in pregnant women in the United States: NHANES 2003-2004.	multi-group	exposure assessment	(Woodruff et al. 2011)

Publication title	Chemical group	Analysis category	Reference
Population variability of phthalate metabolites and bisphenol A concentrations in spot urine samples versus 24- or 48-h collections.	multi-group	exposure assessment	(Christensen et al. 2012)
Social disparities in exposures to bisphenol A and polyfluoroalkyl chemicals: a cross-sectional study within NHANES 2003-2006.	multi-group	exposure assessment	(Nelson et al. 2012)
A method to screen U.S. environmental biomonitoring data for race/ethnicity and income-related disparity.	multi-group	exposure assessment	(Belova et al. 2013)
Associations between socioeconomic status and environmental toxicant concentrations in adults in the USA: NHANES 2001-2010.	multi-group	exposure assessment	(Tyrrell et al. 2013)
High-throughput models for exposure-based chemical prioritization in the ExpoCast project.	multi-group	exposure assessment	(Wambaugh et al. 2013)
Association between serum concentrations of persistent organic pollutants and insulin resistance among nondiabetic adults: results from the National Health and Nutrition Examination Survey 1999-2002.	multi-group	health association	(Lee et al. 2007a)
Relationship between serum concentrations of persistent organic pollutants and the prevalence of metabolic syndrome among non-diabetic adults: results from the National Health and Nutrition Examination Survey 1999-2002.	multi-group	health association	(Lee et al. 2007b)
Polychlorinated biphenyls, lead, and mercury are associated with liver disease in American adults: NHANES 2003-2004.	multi-group	health association	(Cave et al. 2010)

Publication title	Chemical group	Analysis category	Reference
Relationship between urinary phthalate and bisphenol A concentrations and serum thyroid measures in U.S. adults and adolescents from the National Health and Nutrition Examination Survey (NHANES) 2007-2008.	multi-group	health association	(Meeker and Ferguson 2011)
Exposures to endocrine-disrupting chemicals and age of menarche in adolescent girls in NHANES (2003-2008).	multi-group	health association	(Buttke et al. 2012)
Systematic evaluation of environmental factors: persistent pollutants and nutrients correlated with serum lipid levels.	multi-group	health association	(Patel et al. 2012)
Investigation of maternal environmental exposures in association with self-reported preterm birth.	multi-group	health association	(Patel et al. 2014)
Persistent organic pollutants and hyperuricemia in the U.S. general population.	multi-group	health association	(Lee et al. 2013)
Polychlorinated biphenyls, mercury, and antinuclear antibody positivity, NHANES 2003-2004.	multi-group	health association	(Gallagher et al. 2013a)
Systematic evaluation of environmental and behavioural factors associated with all-cause mortality in the United States National Health and Nutrition Examination Survey.	multi-group	health association	(Patel et al. 2013)
Human consumption of methyleugenol and its elimination from serum.	other	exposure assessment	(Schecter et al. 2004)
Perchlorate exposure of the US Population, 2001-2002.	other	exposure assessment	(Blount et al. 2007)

Publication title	Chemical group	Analysis category	Reference
Drinking water contribution to aggregate perchlorate intake of reproductive-age women in the United States estimated by dietary intake simulation and analysis of urinary excretion data.	other	exposure assessment	(Mendez et al. 2010)
Exposure of the U.S. population to acrylamide in the National Health and Nutrition Examination Survey 2003-2004.	other	exposure assessment	(Vesper et al. 2010)
Direct measurement of perchlorate exposure biomarkers in a highly exposed population: a pilot study.	other	exposure assessment	(English et al. 2011)
Estimating perchlorate exposure from food and tap water based on US biomonitoring and occurrence data.	other	exposure assessment	(Huber et al. 2011)
Using a physiologically based pharmacokinetic model to link urinary biomarker concentrations to dietary exposure of perchlorate.	other	exposure assessment	(YC Yang et al. 2012)
Among 10 sociodemographic and lifestyle variables, smoking is strongly associated with biomarkers of acrylamide exposure in a representative sample of the U.S. Population.	other	exposure assessment	(Vesper et al. 2013)
Urinary perchlorate as a measure of dietary and drinking water exposure in a representative sample of the United States population 2001-2008.	other	exposure assessment	(Lau et al. 2013)
Urinary perchlorate and thyroid hormone levels in adolescent and adult men and women living in the United States.	other	health association	(Blount et al. 2006)

Publication title	Chemical group	Analysis category	Reference
Comparison of 1-hydroxypyrene exposure in the US population with that in occupational exposure studies.	PAHs	exposure assessment	(Huang et al. 2004)
Levels of 1-hydroxypyrene and other monohydroxy polycyclic aromatic hydrocarbons in children: a study based on U.S. reference range values.	PAHs	exposure assessment	(Huang et al. 2006)
Reference range levels of polycyclic aromatic hydrocarbons in the US population by measurement of urinary monohydroxy metabolites.	PAHs	exposure assessment	(Grainger et al. 2006)
Concentration and profile of 22 urinary polycyclic aromatic hydrocarbon metabolites in the US population.	PAHs	exposure assessment	(Li et al. 2008)
Involuntary tobacco smoke exposure and urinary levels of polycyclic aromatic hydrocarbons in the United States, 1999 to 2002.	PAHs	exposure assessment	(Suwan-ampai et al. 2009)
Polycyclic aromatic hydrocarbon metabolite levels and pediatric allergy and asthma in an inner-city cohort.	PAHs	exposure assessment	(Miller et al. 2010)
Association of urinary polycyclic aromatic hydrocarbons and serum C-reactive protein.	PAHs	health association	(Everett et al. 2010)
Studying associations between urinary metabolites of polycyclic aromatic hydrocarbons (PAHs) and cardiovascular diseases in the United States.	PAHs	health association	(XH Xu et al. 2010)

Publication title	Chemical group	Analysis category	Reference
Naphthalene biomarkers and relationship with hemoglobin and hematocrit in White, Black, and Hispanic adults: results from the 2003-2004 National Health and Nutrition Examination Survey.	PAHs	health association	(Sudakin et al. 2013)
Studying the effects of polycyclic aromatic hydrocarbons on peripheral arterial disease in the United States.	PAHs	health association	(X Xu et al. 2013)
Assessment of human exposure and human health effects after indoor application of methyl parathion in Lorain County, Ohio, 1995-1996.	pesticides	exposure assessment	(Rubin et al. 2002)
Investigation of the relation between self-reported food consumption and household chemical exposures with urinary levels of selected nonpersistent pesticides.	pesticides	exposure assessment	(Kieszak et al. 2002)
Concentrations of dialkyl phosphate metabolites of organophosphorus pesticides in the U.S. population.	pesticides	exposure assessment	(Barr et al. 2004)
Estimating pesticide dose from urinary pesticide concentration data by creatinine correction in the Third National Health and Nutrition Examination Survey (NHANES-III).	pesticides	exposure assessment	(Mage et al. 2004)
Biologic monitoring to characterize organophosphorus pesticide exposure among children and workers: an analysis of recent studies in Washington State.	pesticides	exposure assessment	(Fenske et al. 2005)
Concentrations of selective metabolites of organophosphorus pesticides in the United States population.	pesticides	exposure assessment	(Barr et al. 2005)

Publication title	Chemical group	Analysis category	Reference
Organophosphate urinary metabolite levels during pregnancy and after delivery in women living in an agricultural community.	pesticides	exposure assessment	(Bradman et al. 2005)
Investigation of job-related pesticide exposure in the Third National Health and Nutrition Examination Survey.	pesticides	exposure assessment	(Allen et al. 2006)
Merging models and biomonitoring data to characterize sources and pathways of human exposure to organophosphorus pesticides in the Salinas Valley of California.	pesticides	exposure assessment	(McKone et al. 2007)
Diet and nondiet predictors of urinary 3-phenoxybenzoic acid in NHANES 1999-2002.	pesticides	exposure assessment	(Riederer et al. 2008)
Evaluating cumulative organophosphorus pesticide body burden of children: a national case study.	pesticides	exposure assessment	(Payne-Sturges et al. 2009)
Comparison of current-use pesticide and other toxicant urinary metabolite levels among pregnant women in the CHAMACOS cohort and NHANES.	pesticides	exposure assessment	(Castorina et al. 2010)
Organophosphorus and pyrethroid insecticide urinary metabolite concentrations in young children living in a southeastern United States city.	pesticides	exposure assessment	(Naeher et al. 2010)
Urinary concentrations of metabolites of pyrethroid insecticides in the general U.S. population: National Health and Nutrition Examination Survey 1999-2002.	pesticides	exposure assessment	(Barr et al. 2010)

Publication title	Chemical group	Analysis category	Reference
Urinary concentrations of dialkylphosphate metabolites of organophosphorus pesticides: National Health and Nutrition Examination Survey 1999-2004.	pesticides	exposure assessment	(Barr et al. 2011)
A pharmacokinetic model of cis- and trans-permethrin disposition in rats and humans with aggregate exposure application.	pesticides	exposure assessment	(Tornero-Velez et al. 2012)
Have regulatory efforts to reduce organophosphorus insecticide exposures been effective?	pesticides	exposure assessment	(Clune et al. 2012)
Associations of serum concentrations of organochlorine pesticides with breast cancer and prostate cancer in U.S. adults.	pesticides	health association	(X Xu et al. 2010)
Attention-deficit/hyperactivity disorder and urinary metabolites of organophosphate pesticides.	pesticides	health association	(Bouchard et al. 2010)
The association between urinary concentrations of dichlorophenol pesticides and obesity in children.	pesticides	health association	(Twum and Wei 2011)
Urinary trichlorophenol levels and increased risk of attention deficit hyperactivity disorder among US school-aged children.	pesticides	health association	(Xu et al. 2011)
Association between urinary 3, 5, 6-trichloro-2-pyridinol, a metabolite of chlorpyrifos and chlorpyrifos-methyl, and serum T4 and TSH in NHANES 1999-2002.	pesticides	health association	(Fortenberry et al. 2012)
Associations between organochlorine pesticides and vitamin D deficiency in the U.S. population.	pesticides	health association	(JH Yang et al. 2012)

Publication title	Chemical group	Analysis category	Reference
Dichlorophenol-containing pesticides and allergies: results from the US National Health and Nutrition Examination Survey 2005-2006.	pesticides	health association	(Jerschow et al. 2012)
Perfluorochemicals in pooled serum samples from United States residents in 2001 and 2002.	PFCs	exposure assessment	(Calafat et al. 2006)
Polyfluoroalkyl chemicals in the U.S. population: data from the National Health and Nutrition Examination Survey (NHANES) 2003-2004 and comparisons with NHANES 1999-2000.	PFCs	exposure assessment	(Calafat et al. 2007b)
Serum concentrations of 11 polyfluoroalkyl compounds in the U.S. population: data from the National Health and Nutrition Examination Survey (NHANES).	PFCs	exposure assessment	(Calafat et al. 2007a)
Polyfluoroalkyl compounds in pooled sera from children participating in the National Health and Nutrition Examination Survey 2001-2002.	PFCs	exposure assessment	(Kato et al. 2009)
Important issues related to using pooled samples for environmental chemical biomonitoring.	PFCs	exposure assessment	(Caudill 2011)
Trends in exposure to polyfluoroalkyl chemicals in the U.S. Population: 1999-2008.	PFCs	exposure assessment	(Kato et al. 2011)
Effect of pregnancy on the levels of selected perfluoroalkyl compounds for females aged 17-39 years: data from National Health and Nutrition Examination Survey 2003-2008.	PFCs	exposure assessment	(Jain 2013)

Publication title	Chemical group	Analysis category	Reference
Association between serum perfluorooctanoic acid (PFOA) and thyroid disease in the U.S. National Health and Nutrition Examination Survey.	PFCs	health association	(Melzer et al. 2010a)
Exposure to polyfluoroalkyl chemicals and attention deficit/hyperactivity disorder in U.S. children 12-15 years of age.	PFCs	health association	(Hoffman et al. 2010)
Exposure to polyfluoroalkyl chemicals and cholesterol, body weight, and insulin resistance in the general U.S. population.	PFCs	health association	(Nelson et al. 2010)
Investigation of the associations between low-dose serum perfluorinated chemicals and liver enzymes in US adults.	PFCs	health association	(Lin et al. 2010)
Perfluorooctanoic acid exposure is associated with elevated homocysteine and hypertension in US adults.	PFCs	health association	(JY Min et al. 2012)
Association between serum perfluorinated chemicals and thyroid function in U.S. adults: the National Health and Nutrition Examination Survey 2007-2010.	PFCs	health association	(Wen et al. 2013)
Positive association between perfluoroalkyl chemicals and hyperuricemia in children.	PFCs	health association	(Geiger et al. 2013)
Medications as a source of human exposure to phthalates.	phthalates	exposure assessment	(Hauser et al. 2004)
Urinary levels of seven phthalate metabolites in the U.S. population from the National Health and Nutrition Examination Survey (NHANES) 1999-2000.	phthalates	exposure assessment	(Silva et al. 2004)

Publication title	Chemical group	Analysis category	Reference
Internal phthalate exposure over the last two decades-a retrospective human biomonitoring study.	phthalates	exposure assessment	(Wittassek et al. 2007)
Medications as a potential source of exposure to phthalates in the U.S. population.	phthalates	exposure assessment	(Hernandez-Diaz et al. 2009)
A simple pharmacokinetic model to characterize exposure of Americans to di-2-ethylhexyl phthalate.	phthalates	exposure assessment	(Lorber et al. 2010)
Dietary intake is associated with phthalate body burden in a nationally representative sample.	phthalates	exposure assessment	(Colacino et al. 2010)
Selecting adequate exposure biomarkers of diisononyl and diisodecyl phthalates: data from the 2005-2006 National Health and Nutrition Examination Survey.	phthalates	exposure assessment	(Calafat et al. 2011)
Socioeconomic factors and phthalate metabolite concentrations among United States women of reproductive age.	phthalates	exposure assessment	(Kobrosly et al. 2012)
Concentrations of urinary phthalate metabolites are associated with increased waist circumference and insulin resistance in adult U.S. males.	phthalates	health association	(Stahlhut et al. 2007)
Urinary phthalate metabolites in relation to biomarkers of inflammation and oxidative stress: NHANES 1999-2006.	phthalates	health association	(Ferguson et al. 2011)
Phthalate exposure and allergy in the U.S. population: results from NHANES 2005-2006.	phthalates	health association	(Hoppin et al. 2013)

Publication title	Chemical group	Analysis category	Reference
Race/ethnicity-specific associations of urinary phthalates with childhood body mass in a nationally representative sample.	phthalates	health association	(Trasande et al. 2013a)
Urinary phthalates are associated with higher blood pressure in childhood.	phthalates	health association	(Trasande et al. 2013c)
Urine phthalate concentrations are higher in people with stroke: United States National Health and Nutrition Examination Surveys (NHANES), 2001-2004.	phthalates	health association	(Shiue 2013)
Reference range concentrations of N-acetyl-S-(2-hydroxyethyl)-L-cysteine, a common metabolite of several volatile organic compounds, in the urine of adults in the United States.	VOCs	exposure assessment	(Calafat et al. 1999)
Environmental exposure of commuters in Mexico City to volatile organic compounds as assessed by blood concentrations, 1998.	VOCs	exposure assessment	(Lemire et al. 2004)
Computational toxicology of chloroform: reverse dosimetry using Bayesian inference, Markov chain Monte Carlo simulation, and human biomonitoring data.	VOCs	exposure assessment	(Lyons et al. 2008)
Relationships between levels of volatile organic compounds in air and blood from the general population.	VOCs	exposure assessment	(Lin et al. 2008)
Public health interpretation of trihalomethane blood levels in the United States: NHANES 1999-2004.	VOCs	exposure assessment	(Lakind et al. 2010)
Impact of cigarette smoking on volatile organic compound (VOC) blood levels in the U.S. population: NHANES 2003-2004.	VOCs	exposure assessment	(Chambers et al. 2011)

Publication title	Chemical group	Analysis category	Reference
Blood/air distribution of volatile organic compounds (VOCs) in a nationally representative sample.	VOCs	exposure assessment	(Jia et al. 2012)
Translational research to develop a human PBPK models tool kit-volatile organic compounds (VOCs).	VOCs	exposure assessment	(Mumtaz et al. 2012)
Volatile organic compounds and pulmonary function in the Third National Health and Nutrition Examination Survey, 1988-1994.	VOCs	health association	(Elliott et al. 2006)

BFRs, brominated flame retardants; PAHs, polycyclic aromatic hydrocarbons; PCBs, polychlorinated biphenyls; PFCs, perfluorinated compounds; VOCs, volatile organic compounds.

References

Adams SV, Passarelli MN, Newcomb PA. 2012. Cadmium exposure and cancer mortality in the Third National Health and Nutrition Examination Survey cohort. Occup Environ Med 69:153-156.

Allen BC, Hack CE, Clewell HJ. 2007. Use of markov chain monte carlo analysis with a physiologically-based pharmacokinetic model of methylmercury to estimate exposures in US women of childbearing age. Risk Anal 27:947-959.

Allen RH, Mage DT, Gondy G, Kodali A, Christensen C, Coble J, et al. 2006. Investigation of job-related pesticide exposure in the Third National Health and Nutrition Examination Survey. Arch Environ Occup Health 61:75-86.

Amster ED, Cho JI, Christiani D. 2011. Urine arsenic concentration and obstructive pulmonary disease in the U.S. population. J Toxicol Environ Health A 74:716-727.

Apostolou A, Garcia-Esquinas E, Fadrowski JJ, McLain P, Weaver VM, Navas-Acien A. 2012. Secondhand tobacco smoke: A source of lead exposure in US children and adolescents. Am J Public Health 102:714-722.

Arora M, Weuve J, Schwartz J, Wright RO. 2009. Association of environmental cadmium exposure with periodontal disease in U.S. adults. Environ Health Perspect 117:739-744.

Ballew C, Khan LK, Kaufmann R, Mokdad A, Miller DT, Gunter EW. 1999. Blood lead concentration and children's anthropometric dimensions in the Third National Health and Nutrition Examination Survey (NHANES III), 1988-1994. J Pediatr 134:623-630.

Barr DB, Bravo R, Weerasekera G, Caltabiano LM, Whitehead RD, Jr., Olsson AO, et al. 2004. Concentrations of dialkyl phosphate metabolites of organophosphorus pesticides in the U.S. population. Environ Health Perspect 112:186-200.

Barr DB, Allen R, Olsson AO, Bravo R, Caltabiano LM, Montesano A, et al. 2005. Concentrations of selective metabolites of organophosphorus pesticides in the United States population. Environ Res 99:314-326.

Barr DB, Olsson AO, Wong LY, Udunka S, Baker SE, Whitehead RD, et al. 2010. Urinary concentrations of metabolites of pyrethroid insecticides in the general U.S. population: National Health and Nutrition Examination Survey 1999-2002. Environ Health Perspect 118:742-748.

Barr DB, Wong LY, Bravo R, Weerasekera G, Odetokun M, Restrepo P, et al. 2011. Urinary concentrations of dialkylphosphate metabolites of organophosphorus pesticides: National Health and Nutrition Examination Survey 1999-2004. Int J Environ Res Public Health 8:3063-3098.

Belova A, Greco SL, Riederer AM, Olsho LEW, Corrales MA. 2013. A method to screen U.S. environmental biomonitoring data for race/ethnicity and income-related disparity. Environ Health 12:114.

Bernard SM, McGeehin MA. 2003. Prevalence of blood lead levels >or= 5 micro g/dl among US children 1 to 5 years of age and socioeconomic and demographic factors associated with blood of lead levels 5 to 10 micro g/dl, Third National Health and Nutrition Examination Survey, 1988-1994. Pediatrics 112:1308-1313.

Bhandari R, Xiao J, Shankar A. 2013. Urinary bisphenol A and obesity in U.S. children. Am J Epidemiol 177:1263-1270.

Blount BC, Pirkle JL, Osterloh JD, Valentin-Blasini L, Caldwell KL. 2006. Urinary perchlorate and thyroid hormone levels in adolescent and adult men and women living in the United States. Environ Health Perspect 114:1865-1871.

Blount BC, Valentin-Blasini L, Osterloh JD, Mauldin JP, Pirkle JL. 2007. Perchlorate exposure of the US population, 2001-2002. J Expo Sci Environ Epidemiol 17:400-407.

Bouchard MF, Bellinger DC, Weuve J, Matthews-Bellinger J, Gilman SE, Wright RO, et al. 2009. Blood lead levels and major depressive disorder, panic disorder, and generalized anxiety disorder in US young adults. Arch Gen Psychiatry 66:1313-1319.

Bouchard MF, Bellinger DC, Wright RO, Weisskopf MG. 2010. Attention-deficit/hyperactivity disorder and urinary metabolites of organophosphate pesticides. Pediatrics 125:E1270-E1277.

Bradman A, Eskenazi B, Barr DB, Bravo R, Castorina R, Chevrier J, et al. 2005. Organophosphate urinary metabolite levels during pregnancy and after delivery in women living in an agricultural community. Environ Health Perspect 113:1802-1807.

Braun JM, Kahn RS, Froehlich T, Auinger P, Lanphear BP. 2006. Exposures to environmental toxicants and attention deficit hyperactivity disorder in U.S. children. Environ Health Perspect 114:1904-1909.

Braun JM, Froehlich TE, Daniels JL, Dietrich KN, Hornung R, Auinger P, et al. 2008. Association of environmental toxicants and conduct disorder in U.S. children: NHANES 2001-2004. Environ Health Perspect 116:956-962.

Buettner C, Mukamal KJ, Gardiner P, Davis RB, Phillips RS, Mittleman MA. 2009. Herbal supplement use and blood lead levels of United States adults. J Gen Intern Med 24:1175-1182.

Buttke DE, Sircar K, Martin C. 2012. Exposures to endocrine-disrupting chemicals and age of menarche in adolescent girls in NHANES (2003-2008). Environ Health Perspect 120:1613-1618.

Calafat AM, Barr DB, Pirkle JL, Ashley DL. 1999. Reference range concentrations of n-acetyl-s-(2-hydroxyethyl)-l-cysteine, a common metabolite of several volatile organic compounds, in the urine of adults in the United States. J Expo Anal Environ Epidemiol 9:336-342.

Calafat AM, Kuklenyik Z, Caudill SP, Reidy JA, Needham LL. 2006. Perfluorochemicals in pooled serum samples from United States residents in 2001 and 2002. Environ Sci Technol 40:2128-2134.

Calafat AM, Kuklenyik Z, Reidy JA, Caudill SP, Tully JS, Needham LL. 2007a. Serum concentrations of 11 polyfluoroalkyl compounds in the U.S. population: Data from the National Health and Nutrition Examination Survey (NHANES). Environ Sci Technol 41:2237-2242.

Calafat AM, Wong LY, Kuklenyik Z, Reidy JA, Needham LL. 2007b. Polyfluoroalkyl chemicals in the U.S. population: Data from the National Health and Nutrition Examination Survey (NHANES) 2003-2004 and comparisons with NHANES 1999-2000. Environ Health Perspect 115:1596-1602.

Calafat AM, Wong LY, Ye X, Reidy JA, Needham LL. 2008a. Concentrations of the sunscreen agent benzophenone-3 in residents of the United States: National Health and Nutrition Examination Survey 2003-2004. Environ Health Perspect 116:893-897.

Calafat AM, Ye X, Wong LY, Reidy JA, Needham LL. 2008b. Urinary concentrations of triclosan in the U.S. population: 2003-2004. Environ Health Perspect 116:303-307.

Calafat AM, Ye X, Wong LY, Reidy JA, Needham LL. 2008c. Exposure of the U.S. population to bisphenol A and 4-tertiary-octylphenol: 2003-2004. Environ Health Perspect 116:39-44.

Calafat AM, Ye X, Wong LY, Bishop AM, Needham LL. 2010. Urinary concentrations of four parabens in the U.S. population: NHANES 2005-2006. Environ Health Perspect 118:679-685.

Calafat AM, Wong LY, Silva MJ, Samandar E, Preau JL, Jia LT, et al. 2011. Selecting adequate exposure biomarkers of diisononyl and diisodecyl phthalates: Data from the 2005-2006 National Health and Nutrition Examination Survey. Environ Health Perspect 119:50-55.

Caldwell KL, Jones RL, Verdon CP, Jarrett JM, Caudill SP, Osterloh JD. 2009a. Levels of urinary total and speciated arsenic in the US population: National Health and Nutrition Examination Survey 2003-2004. J Expo Sci Environ Epidemiol 19:59-68.

Caldwell KL, Mortensen ME, Jones RL, Caudill SP, Osterloh JD. 2009b. Total blood mercury concentrations in the U.S. population: 1999-2006. Int J Hyg Environ Health 212:588-598.

Carrington CD, Bolger MP. 2002. An exposure assessment for methylmercury from seafood for consumers in the United States. Risk Anal 22:689-699.

Carrington CD, Montwill B, Bolger PM. 2004. An intervention analysis for the reduction of exposure to methylmercury from the consumption of seafood by women of child-bearing age. Regul Toxicol Pharm 40:272-280.

Carwile JL, Michels KB. 2011. Urinary bisphenol A and obesity: NHANES 2003-2006. Environ Res 111:825-830.

Castorina R, Bradman A, Fenster L, Barr DB, Bravo R, Vedar MG, et al. 2010. Comparison of current-use pesticide and other toxicant urinary metabolite levels among pregnant women in the Chamacos cohort and NHANES. Environ Health Perspect 118:856-863.

Caudill SP. 2011. Important issues related to using pooled samples for environmental chemical biomonitoring. Stat Med 30:515-521.

Caudill SP. 2012. Use of pooled samples from the National Health and Nutrition Examination Survey. Stat Med 31:3269-3277.

Cave M, Appana S, Patel M, Falkner KC, McClain CJ, Brock G. 2010. Polychlorinated biphenyls, lead, and mercury are associated with liver disease in American adults: NHANES 2003-2004. Environ Health Perspect 118:1735-1742.

CDC. 2000. Blood lead levels in young children-United States and selected states, 1996-1999. MMWR Morb Mortal Wkly Rep 49:1133-1137.

CDC. 2001. Blood and hair mercury levels in young children and women of childbearing age-United States, 1999. MMWR Morb Mortal Wkly Rep 50:140-143.

CDC. 2004. Blood mercury levels in young children and childbearing-aged women-United States, 1999-2002. MMWR Morb Mortal Wkly Rep 53:1018-1020.

CDC. 2005. Blood lead levels-United States, 1999-2002. MMWR Morb Mortal Wkly Rep 54:513-516.

Chambers DM, Ocariz JM, McGuirk MF, Blount BC. 2011. Impact of cigarette smoking on volatile organic compound (VOC) blood levels in the U.S. population: NHANES 2003-2004. Environ Int 37:1321-1328.

Chen AM, Kim SS, Chung E, Dietrich KN. 2013. Thyroid hormones in relation to lead, mercury, and cadmium exposure in the National Health and Nutrition Examination Survey, 2007-2008. Environ Health Perspect 121:181-186.

Chen Q, Jiang X, Hedgeman E, Knutson K, Gillespie B, Hong B, et al. 2013. Estimation of age- and sex-specific background human serum concentrations of PCDDs, PCDFs, and PCBs in the UMDES and NHANES populations. Chemosphere 91:817-823.

Choi YH, Hu H, Mukherjee B, Miller J, Park SK. 2012. Environmental cadmium and lead exposures and hearing loss in U.S. adults: The National Health and Nutrition Examination Survey, 1999 to 2004. Environ Health Perspect 120:1544-1550.

Choudhury H, Harvey T, Thayer WC, Lockwood TF, Stiteler WM, Goodrum PE, et al. 2001. Urinary cadmium elimination as a biomarker of exposure for evaluating a cadmium dietary exposure-biokinetics model. J Toxicol Environ Health A 63:321-350.

Christensen KLY, Lorber M, Koch HM, Kolossa-Gehring M, Morgan MK. 2012. Population variability of phthalate metabolites and bisphenol A concentrations in spot urine samples versus 24-or 48-h collections. J Expo Sci Environ Epidemiol 22:632-640.

Christensen KLY. 2013. Metals in blood and urine, and thyroid function among adults in the United States 2007-2008. Int J Hyg Environ Health 216:624-632.

Ciesielski T, Weuve J, Bellinger DC, Schwartz J, Lanphear B, Wright RO. 2012. Cadmium exposure and neurodevelopmental outcomes in U.S. children. Environ Health Perspect 120:758-763.

Ciesielski T, Bellinger DC, Schwartz J, Hauser R, Wright RO. 2013. Associations between cadmium exposure and neurocognitive test scores in a cross-sectional study of US adults. Environ Health 12:13.

Clayton EMR, Todd M, Dowd JB, Aiello AE. 2011. The impact of bisphenol A and triclosan on immune parameters in the U.S. population, NHANES 2003-2006. Environ Health Perspect 119:390-396.

Clune AL, Ryan PB, Barr DB. 2012. Have regulatory efforts to reduce organophosphorus insecticide exposures been effective? Environ Health Perspect 120:521-525.

Colacino JA, Harris TR, Schecter A. 2010. Dietary intake is associated with phthalate body burden in a nationally representative sample. Environ Health Perspect 118:998-1003.

Crinnion WJ. 2010. The CDC fourth national report on human exposure to environmental chemicals: What it tells us about our toxic burden and how it assist environmental medicine physicians. Altern Med Rev 15:101-109.

Davis MA, Mackenzie TA, Cottingham KL, Gilbert-Diamond D, Punshon T, Karagas MR. 2012. Rice consumption and urinary arsenic concentrations in U.S. children. Environ Health Perspect 120:1418-1424.

Dixon SL, Gaitens JM, Jacobs DE, Strauss W, Nagaraja J, Pivetz T, et al. 2009. Exposure of U.S. children to residential dust lead, 1999-2004: II. The contribution of lead-contaminated dust to children's blood lead levels. Environ Health Perspect 117:468-474.

Dye BA, Hirsch R, Brody DJ. 2002. The relationship between blood lead levels and periodontal bone loss in the United States, 1988-1994. Environ Health Perspect 110:997-1002.

Dye BA, Schober SE, Dillon CF, Jones RL, Fryar C, McDowell M, et al. 2005. Urinary mercury concentrations associated with dental restorations in adult women aged 16-49 years: United States, 1999-2000. Occup Environ Med 62:368-375.

Elliott L, Longnecker MP, Kissling GE, London SJ. 2006. Volatile organic compounds and pulmonary function in the Third National Health and Nutrition Examination Survey, 1988-1994. Environ Health Perspect 114:1210-1214.

Eng DS, Lee JM, Gebremariam A, Meeker JD, Peterson K, Padmanabhan V. 2013. Bisphenol A and chronic disease risk factors in US children. Pediatrics 132:e637-645.

English P, Blount B, Wong M, Copan L, Olmedo L, Patton S, et al. 2011. Direct measurement of perchlorate exposure biomarkers in a highly exposed population: A pilot study. PLoS One 6:e17015.

Everett CJ, King DE, Player MS, Matheson EM, Post RE, Mainous AG, 3rd. 2010. Association of urinary polycyclic aromatic hydrocarbons and serum c-reactive protein. Environ Res 110:79-82.

Fadrowski JJ, Navas-Acien A, Tellez-Plaza M, Guallar E, Weaver VM, Furth SL. 2010. Blood lead level and kidney function in US adolescents: The Third National Health and Nutrition Examination Survey. Arch Intern Med 170:75-82.

Faramawi MF, Liu YC, Caffrey JL, Lin YS, Gandhi S, Singh KP. 2012. The association between urinary cadmium and frontal t wave axis deviation in the US adults. Int J Hyg Environ Health 215:406-410.

Fenske RA, Lu CS, Curl CL, Shirai JH, Kissel JC. 2005. Biologic monitoring to characterize organophosphorus pesticide exposure among children and workers: An analysis of recent studies in Washington State. Environ Health Perspect 113:1651-1657.

Ferguson KK, Loch-Caruso R, Meeker JD. 2011. Urinary phthalate metabolites in relation to biomarkers of inflammation and oxidative stress: NHANES 1999-2006. Environ Res 111:718-726.

Ferraro PM, Costanzi S, Naticchia A, Sturniolo A, Gambaro G. 2010. Low level exposure to cadmium increases the risk of chronic kidney disease: Analysis of the NHANES 1999-2006. BMC public health 10:304.

Ferraro PM, Sturniolo A, Naticchia A, D'Alonzo S, Gambaro G. 2012. Temporal trend of cadmium exposure in the United States population suggests gender specificities. Intern Med J 42:691-697.

Ferriby LL, Knutsen JS, Harris M, Unice KM, Scott P, Nony P, et al. 2007. Evaluation of PCDD/F and dioxin-like PCB serum concentration data from the 2001-2002 National Health and Nutrition Examination Survey of the United States population. J Expo Sci Environ Epidemiol 17:358-371.

Ford ES. 1999. Serum magnesium and ischaemic heart disease: Findings from a national sample of US adults. Int J Epidemiol 28:645-651.

Ford ES. 2000. Serum copper concentration and coronary heart disease among US adults. Am J Epidemiol 151:1182-1188.

Fortenberry GZ, Hu H, Turyk M, Barr DB, Meeker JD. 2012. Association between urinary 3, 5, 6-trichloro-2-pyridinol, a metabolite of chlorpyrifos and chlorpyrifos-methyl, and serum T4 and TSH in NHANES 1999-2002. Sci Total Environ 424:351-355.

Fraser AJ, Webster TF, McClean MD. 2009. Diet contributes significantly to the body burden of PBDEs in the general US population. Environ Health Perspect 117:1520-1525.

Froehlich TE, Lanphear BP, Auinger P, Hornung R, Epstein JN, Braun J, et al. 2009. Association of tobacco and lead exposures with attention-deficit/hyperactivity disorder. Pediatrics 124:e1054-1063.

Gallagher CM, Kovach JS, Meliker JR. 2008. Urinary cadmium and osteoporosis in U.S. women >or= 50 years of age: NHANES 1988-1994 and 1999-2004. Environ Health Perspect 116:1338-1343.

Gallagher CM, Chen JJ, Kovach JS. 2010a. Environmental cadmium and breast cancer risk. Aging (Albany NY) 2:804-814.

Gallagher CM, Moonga BS, Kovach JS. 2010b. Cadmium, follicle-stimulating hormone, and effects on bone in women age 42-60 years, NHANES III. Environ Res 110:105-111.

Gallagher CM, Meliker JR. 2011. Total blood mercury, plasma homocysteine, methylmalonic acid and folate in US children aged 3-5 years, NHANES 1999-2004. Sci Total Environ 409:1399-1405.

Gallagher CM, Smith DM, Meliker JR. 2011. Total blood mercury and serum measles antibodies in US children, NHANES 2003-2004. Sci Total Environ 410-411:65-71.

Gallagher CM, Meliker JR. 2012. Mercury and thyroid autoantibodies in U.S. women, NHANES 2007-2008. Environ Int 40:39-43.

Gallagher CM, McElroy AE, Smith DM, Golightly MG, Meliker JR. 2013a. Polychlorinated biphenyls, mercury, and antinuclear antibody positivity, NHANES 2003-2004. Int J Hyg Environ Health 216:721-727.

Gallagher CM, Smith DM, Golightly MG, Meliker JR. 2013b. Total blood mercury and rubella antibody concentrations in US children aged 6-11 years, NHANES 2003-2004. Sci Total Environ 442:48-55.

Geiger SD, Xiao J, Shankar A. 2013. Positive association between perfluoroalkyl chemicals and hyperuricemia in children. Am J Epidemiol 177:1255-1262.

Gollenberg AL, Hediger ML, Lee PA, Himes JH, Louis GMB. 2010. Association between lead and cadmium and reproductive hormones in peripubertal U.S. girls. Environ Health Perspect 118:1782-1787.

Golub NI, Winters PC, van Wijngaarden E. 2010. A population-based study of blood lead levels in relation to depression in the United States. Int Arch Occup Environ Health 83:771-777.

Goodrich JM, Wang Y, Gillespie B, Werner R, Franzblau A, Basu N. 2013. Methylmercury and elemental mercury differentially associate with blood pressure among dental professionals. Int J Hyg Environ Health 216:195-201.

Grainger J, Huang W, Patterson DG, Jr., Turner WE, Pirkle J, Caudill SP, et al. 2006. Reference range levels of polycyclic aromatic hydrocarbons in the US population by measurement of urinary monohydroxy metabolites. Environ Res 100:394-423.

Grosse SD, Matte TD, Schwartz J, Jackson RJ. 2002. Economic gains resulting from the reduction in children's exposure to lead in the United States. Environ Health Perspect 110:563-569.

Hauser R, Duty S, Godfrey-Bailey L, Calafat AM. 2004. Medications as a source of human exposure to phthalates. Environ Health Perspect 112:751-753.

Hernandez-Diaz S, Mitchell AA, Kelley KE, Calafat AM, Hauser R. 2009. Medications as a potential source of exposure to phthalates in the US population. Environ Health Perspect 117:185-189.

Hoffman K, Webster TF, Weisskopf MG, Weinberg J, Vieira VM. 2010. Exposure to polyfluoroalkyl chemicals and attention deficit/hyperactivity disorder in U.S. children 12-15 years of age. Environ Health Perspect 118:1762-1767.

Hoppin JA, Jaramillo R, London SJ, Bertelsen RJ, Salo PM, Sandler DP, et al. 2013. Phthalate exposure and allergy in the U.S. population: Results from NHANES 2005-2006. Environ Health Perspect 121:1129-1134.

Hsu JY, Small DS. 2013. Calibrating sensitivity analyses to observed covariates in observational studies. Biometrics 69:803-811.

Huang W, Caudill SP, Grainger J, Needham LL, Patterson DG, Jr. 2006. Levels of 1-hydroxypyrene and other monohydroxy polycyclic aromatic hydrocarbons in children: A study based on U.S. reference range values. Toxicol Lett 163:10-19.

Huang WL, Grainger J, Patterson DG, Turner WE, Caudill SP, Needham LL, et al. 2004. Comparison of 1-hydroxypyrene exposure in the US population with that in occupational exposure studies. Int Arch Occup Environ Health 77:491-498.

Huber DR, Blount BC, Mage DT, Letkiewicz FJ, Kumar A, Allen RH. 2011. Estimating perchlorate exposure from food and tap water based on US biomonitoring and occurrence data. J Expo Sci Environ Epidemiol 21:395-407.

Hyder O, Chung M, Cosgrove D, Herman JM, Li ZP, Firoozmand A, et al. 2013. Cadmium exposure and liver disease among US adults. J Gastrointest Surg 17:1265-1273.

Iqbal S, Muntner P, Batuman V, Rabito FA. 2008. Estimated burden of blood lead levels 5 microg/dl in 1999-2002 and declines from 1988 to 1994. Environ Res 107:305-311.

Jacobs DE, Nevin R. 2006. Validation of a 20-year forecast of US childhood lead poisoning: Updated prospects for 2010. Environ Res 102:352-364.

Jain RB, Wang RY. 2010. Regression models to estimate total polychlorinated biphenyls in the general US population: 2001-2002 and 2003-2004. Chemosphere 79:243-252.

Jain RB, Wang RY. 2011. Association of caffeine consumption and smoking status with the serum concentrations of polychlorinated biphenyls, dioxins, and furans in the general U.S. population: NHANES 2003-2004. J Toxicol Environ Health A 74:1225-1239.

Jain RB. 2013. Effect of pregnancy on the levels of selected perfluoroalkyl compounds for females aged 17-39 years: Data from National Health and Nutrition Examination Survey 2003-2008. J Toxicol Environ Health A 76:409-421.

Jemal A, Graubard BI, Devesa SS, Flegal KM. 2002. The association of blood lead level and cancer mortality among whites in the United States. Environ Health Perspect 110:325-329.

Jerschow E, McGinn AP, de Vos G, Vernon N, Jariwala S, Hudes G, et al. 2012. Dichlorophenol-containing pesticides and allergies: Results from the US National Health and Nutrition Examination Survey 2005-2006. Ann Allergy Asthma Immunol 109:420-425.

Ji JS, Elbaz A, Weisskopf MG. 2013. Association between blood lead and walking speed in the National Health and Nutrition Examination Survey (NHANES 1999-2002). Environ Health Perspect 121:711-716.

Jia C, Yu X, Masiak W. 2012. Blood/air distribution of volatile organic compounds (VOCs) in a nationally representative sample. Sci Total Environ 419:225-232.

Jones L, Parker JD, Mendola P. 2010. Blood lead and mercury levels in pregnant women in the United States, 2003-2008. NCHS data brief:1-8.

Jones MR, Tellez-Plaza M, Sharrett AR, Guallar E, Navas-Acien A. 2011. Urine arsenic and hypertension in US adults: The 2003-2008 National Health and Nutrition Examination Survey. Epidemiology 22:153-161.

Jones RL, Homa DM, Meyer PA, Brody DJ, Caldwell KL, Pirkle JL, et al. 2009. Trends in blood lead levels and blood lead testing among US children aged 1 to 5 years, 1988-2004. Pediatrics 123:e376-e385.

Kato K, Calafat AM, Wong LY, Wanigatunga AA, Caudill SP, Needham LL. 2009. Polyfluoroalkyl compounds in pooled sera from children participating in the National Health and Nutrition Examination Survey 2001-2002. Environ Sci Technol 43:2641-2647.

Kato K, Wong LY, Jia LT, Kuklenyik Z, Calafat AM. 2011. Trends in exposure to polyfluoroalkyl chemicals in the U.S. population: 1999-2008. Environ Sci Technol 45:8037-8045.

Kaufmann RB, Clouse TL, Olson DR, Matte TD. 2000. Elevated blood lead levels and blood lead screening among US children aged one to five years: 1988-1994. Pediatrics 106.

Kieszak SM, Naeher LP, Rubin CS, Needham LL, Backer L, Barr D, et al. 2002. Investigation of the relation between self-reported food consumption and household chemical exposures with urinary levels of selected nonpersistent pesticides. J Expo Anal Environ Epidemiol 12:404-408.

Kobrosly RW, Parlett LE, Stahlhut RW, Barrett ES, Swan SH. 2012. Socioeconomic factors and phthalate metabolite concentrations among United States women of reproductive age. Environ Res 115:11-17.

Koeppe ES, Ferguson KK, Colacino JA, Meeker JD. 2013. Relationship between urinary triclosan and paraben concentrations and serum thyroid measures in NHANES 2007-2008. Sci Total Environ 445:299-305.

Lacher DA, Hughes JP, Carroll MD. 2010. Biological variation of laboratory analytes based on the 1999-2002 National Health and Nutrition Examination Survey. Natl Health Stat Report 1-7.

Lakind JS, Naiman DQ. 2008. Bisphenol A (BPA) daily intakes in the United States: Estimates from the 2003-2004 NHANES urinary BPA data. J Expo Sci Environ Epidemiol 18:608-615.

LaKind JS, Hays SM, Aylward LL, Naiman DQ. 2009. Perspective on serum dioxin levels in the United States: An evaluation of the NHANES data. J Expo Sci Environ Epidemiol 19:435-441.

Lakind JS, Naiman DQ, Hays SM, Aylward LL, Blount BC. 2010. Public health interpretation of trihalomethane blood levels in the United States: NHANES 1999-2004. J Expo Sci Environ Epidemiol 20:255-262.

Lakind JS, Naiman DQ. 2011. Daily intake of bisphenol A and potential sources of exposure: 2005-2006 National Health and Nutrition Examination Survey. J Expo Sci Environ Epidemiol 21:272-279.

LaKind JS, Levesque J, Dumas P, Bryan S, Clarke J, Naiman DQ. 2012. Comparing United States and Canadian population exposures from national biomonitoring surveys: Bisphenol A intake as a case study. J Expo Sci Environ Epidemiol 22:219-226.

Laks DR. 2009. Assessment of chronic mercury exposure within the U.S. population, National Health and Nutrition Examination Survey, 1999-2006. Biometals 22:1103-1114.

Lang IA, Galloway TS, Scarlett A, Henley WE, Depledge M, Wallace RB, et al. 2008. Association of urinary bisphenol A concentration with medical disorders and laboratory abnormalities in adults. J Am Med Assoc 300:1303-1310.

Lanphear BP, Dietrich K, Auinger P, Cox C. 2000. Cognitive deficits associated with blood lead concentrations <10 microg/dl in US children and adolescents. Public Health Rep 115:521-529.

Lau FK, deCastro BR, Mills-Herring L, Tao L, Valentin-Blasini L, Alwis KU, et al. 2013. Urinary perchlorate as a measure of dietary and drinking water exposure in a representative sample of the United States population 2001-2008. J Expo Sci Environ Epidemiol 23:207-214.

Lee DH, Lim JS, Song K, Boo Y, Jacobs DR, Jr. 2006. Graded associations of blood lead and urinary cadmium concentrations with oxidative-stress-related markers in the U.S. population: Results from the Third National Health and Nutrition Examination Survey. Environ Health Perspect 114:350-354.

Lee DH, Lee IK, Jin SH, Steffes M, Jacobs DR, Jr. 2007a. Association between serum concentrations of persistent organic pollutants and insulin resistance among nondiabetic adults: Results from the National Health and Nutrition Examination Survey 1999-2002. Diabetes Care 30:622-628.

Lee DH, Lee IK, Porta M, Steffes M, Jacobs DR. 2007b. Relationship between serum concentrations of persistent organic pollutants and the prevalence of metabolic syndrome among non-diabetic adults: Results from the National Health and Nutrition Examination Survey 1999-2002. Diabetologia 50:1841-1851.

Lee MG, Chun OK, Song WO. 2005. Determinants of the blood lead level of US women of reproductive age. J Am Coll Nutr 24:1-9.

Lee YM, Lee MK, Bae SG, Lee SH, Kim SY, Lee DH. 2012. Association of homocysteine levels with blood lead levels and micronutrients in the US general population. J Prev Med Public Health 45:387-393.

Lee YM, Bae SG, Lee SH, Jacobs DR, Jr., Lee DH. 2013. Persistent organic pollutants and hyperuricemia in the U.S. general population. Atherosclerosis 230:1-5.

Lemire S, Ashley D, Olaya P, Romieu I, Welch S, Meneses-Gonzalez F, et al. 2004. Environmental exposure of commuters in Mexico City to volatile organic compounds as assessed by blood concentrations, 1998. Salud Publica Mex 46:32-38.

Li Z, Sandau CD, Romanoff LC, Caudill SP, Sjodin A, Needham LL, et al. 2008. Concentration and profile of 22 urinary polycyclic aromatic hydrocarbon metabolites in the US population. Environ Res 107:320-331.

Lim JS, Lee DH, Jacobs DR, Jr. 2008. Association of brominated flame retardants with diabetes and metabolic syndrome in the U.S. population, 2003-2004. Diabetes Care 31:1802-1807.

Lin CY, Lin LY, Chiang CK, Wang WJ, Su YN, Hung KY, et al. 2010. Investigation of the associations between low-dose serum perfluorinated chemicals and liver enzymes in US adults. Am J Gastroenterol 105:1354-1363.

Lin YS, Egeghy PP, Rappaport SM. 2008. Relationships between levels of volatile organic compounds in air and blood from the general population. J Expo Sci Environ Epidemiol 18:421-429.

Lorber M, Patterson D, Huwe J, Kahn H. 2009. Evaluation of background exposures of Americans to dioxin-like compounds in the 1990s and the 2000s. Chemosphere 77:640-651.

Lorber M, Angerer J, Koch HM. 2010. A simple pharmacokinetic model to characterize exposure of Americans to di-2-ethylhexyl phthalate. J Expo Sci Environ Epidemiol 20:38-53.

Lustberg M, Silbergeld E. 2002. Blood lead levels and mortality. Arch Intern Med 162:2443-2449.

Lyons MA, Yang RSH, Mayeno AN, Reisfeld B. 2008. Computational toxicology of chloroform: Reverse dosimetry using bayesian inference, markov chain monte carlo simulation, and human biomonitoring data. Environ Health Perspect 116:1040-1046.

Macek MD, Matte TD, Sinks T, Malvitz DM. 2006. Blood lead concentrations in children and method of water fluoridation in the United States, 1988-1994. Environ Health Perspect 114:130-134.

Mage DT, Allen RH, Gondy G, Smith W, Barr DB, Needham LL. 2004. Estimating pesticide dose from urinary pesticide concentration data by creatinine correction in the Third National Health and Nutrition Examination Survey (NHANES-III). J Expo Anal Environ Epidemiol 14:457-465.

Mahaffey KR, Clickner RP, Bodurow CC. 2004. Blood organic mercury and dietary mercury intake: National Health and Nutrition Examination Survey, 1999 and 2000. Environ Health Perspect 112:562-570.

Mahaffey KR. 2005. Mercury exposure: Medical and public health issues. Trans Am Clin Climatol Assoc 116:127-153; discussion 153-124.

Mahaffey KR, Clickner RP, Jeffries RA. 2008. Methylmercury and omega-3 fatty acids: Co-occurrence of dietary sources with emphasis on fish and shellfish. Environ Res 107:20-29.

Mahaffey KR, Clickner RP, Jeffries RA. 2009. Adult women's blood mercury concentrations vary regionally in the United States: Association with patterns of fish consumption (NHANES 1999-2004). Environ Health Perspect 117:47-53.

Mannino DM, Albalak R, Grosse S, Repace J. 2003. Second-hand smoke exposure and blood lead levels in U.S. children. Epidemiology 14:719-727.

Mannino DM, Holguin F, Greves HM, Savage-Brown A, Stock AL, Jones RL. 2004. Urinary cadmium levels predict lower lung function in current and former smokers: Data from the Third National Health and Nutrition Examination Survey. Thorax 59:194-198.

Mannino DM, Homa DM, Matte T, Hernandez-Avila M. 2005. Active and passive smoking and blood lead levels in U.S. adults: Data from the Third National Health and Nutrition Examination Survey. Nicotine Tob Res 7:557-564.

Marchitti SA, LaKind JS, Naiman DQ, Berlin CM, Kenneke JF. 2013. Improving infant exposure and health risk estimates: Using serum data to predict polybrominated diphenyl ether concentrations in breast milk. Environ Sci Technol 47:4787-4795.

McKone TE, Castorina R, Harnly ME, Kuwabara Y, Eskenazi B, Bradman A. 2007. Merging models and biomonitoring data to characterize sources and pathways of human exposure to organophosphorus pesticides in the Salinas Valley of California. Environ Sci Technol 41:3233-3240.

Meeker JD, Ferguson KK. 2011. Relationship between urinary phthalate and bisphenol A concentrations and serum thyroid measures in US adults and adolescents from the National Health and Nutrition Examination Survey (NHANES) 2007-2008. Environ Health Perspect 119:1396-1402.

Melzer D, Rice N, Depledge MH, Henley WE, Galloway TS. 2010a. Association between serum perfluorooctanoic acid (PFOA) and thyroid disease in the US National Health and Nutrition Examination Survey. Environ Health Perspect 118:686-692.

Melzer D, Rice NE, Lewis C, Henley WE, Galloway TS. 2010b. Association of urinary bisphenol A concentration with heart disease: Evidence from NHANES 2003/06. PLoS One 5: e8673

Mendez W, Dederick E, Cohen J. 2010. Drinking water contribution to aggregate perchlorate intake of reproductive-age women in the United States estimated by dietary intake simulation and analysis of urinary excretion data. J Expo Sci Environ Epidemiol 20:288-297.

Mendola P, Brett K, DiBari JN, Pollack AZ, Tandon R, Shenassa ED. 2013. Menopause and lead body burden among US women aged 45-55, NHANES 1999-2010. Environ Res 121:110-113.

Mendy A, Gasana J, Vieira ER. 2012. Urinary heavy metals and associated medical conditions in the US adult population. Int J Environ Health Res 22:105-118.

Menke A, Muntner P, Batuman V, Silbergeld EK, Guallar E. 2006. Blood lead below 0.48 micromol/l (10 microg/dl) and mortality among US adults. Circulation 114:1388-1394.

Menke A, Guallar E, Shiels MS, Rohrmann S, Basaria S, Rifai N, et al. 2008. The association of urinary cadmium with sex steroid hormone concentrations in a general population sample of US adult men. BMC Public Health 8:72.

Menke A, Muntner P, Silbergeld EK, Platz EA, Guallar E. 2009. Cadmium levels in urine and mortality among US adults. Environ Health Perspect 117:190-196.

Meyer PA, Pivetz T, Dignam TA, Homa DM, Schoonover J, Brody D, et al. 2003. Surveillance for elevated blood lead levels among children-United States, 1997-2001. MMWR Surveill Summ 52:1-21.

Mijal RS, Holzman CB. 2010. Blood cadmium levels in women of childbearing age vary by race/ethnicity. Environ Res 110:505-512.

Miller RL, Garfinkel R, Lendor C, Hoepner L, Li Z, Romanoff L, et al. 2010. Polycyclic aromatic hydrocarbon metabolite levels and pediatric allergy and asthma in an inner-city cohort. Pediatr Allergy Immunol 21:260-267.

Min JY, Lee KJ, Park JB, Min KB. 2012. Perfluorooctanoic acid exposure is associated with elevated homocysteine and hypertension in US adults. Occup Environ Med 69:658-662.

Min JY, Min KB. 2013. Association between total blood mercury and exhaled nitric oxide in US adults. Nitric Oxide 29:53-58.

Min KB, Lee KJ, Park JB, Min JY. 2012. Lead and cadmium levels and balance and vestibular dysfunction among adult participants in the National Health and Nutrition Examination Survey (NHANES) 1999-2004. Environ Health Perspect 120:413-417.

Morales LS, Gutierrez P, Escarge JJ. 2005. Demographic and socioeconomic factors associated with blood lead levels among Mexican-American children and adolescents in the United States. Public Health Rep 120:448-454.

Mortensen ME, Wong LY, Osterloh JD. 2011. Smoking status and urine cadmium above levels associated with subclinical renal effects in U.S. adults without chronic kidney disease. Int J Hyg Environ Health 214:305-310.

Moss ME, Lanphear BP, Auinger P. 1999. Association of dental caries and blood lead levels. JAMA 281:2294-2298.

Muennig P, Song XY, Payne-Sturges DC, Gee GC. 2011. Blood and urine levels of long half-life toxicants by nativity among immigrants to the United States. Sci Total Environ 412:109-113.

Mumtaz MM, Ray M, Crowell SR, Keys D, Fisher J, Ruiz P. 2012. Translational research to develop a human pbpk models tool kit-volatile organic compounds (VOCs). J Toxicol Environ Health A 75:6-24.

Muntner P, He J, Vupputuri S, Coresh J, Batuman V. 2003. Blood lead and chronic kidney disease in the general United States population: Results from NHANES III. Kidney Int 63:1044-1050.

Muntner P, Menke A, DeSalvo KB, Rabito FA, Batuman V. 2005. Continued decline in blood lead levels among adults in the United States - the National Health and Nutrition Examination Surveys. Arch Intern Med 165:2155-2161.

Naeher LP, Tulve NS, Egeghy PP, Barr DB, Adetona O, Fortmann RC, et al. 2010. Organophosphorus and pyrethroid insecticide urinary metabolite concentrations in young children living in a southeastern United States city. Sci Total Environ 408:1145-1153.

Nahar MS, Soliman AS, Colacino JA, Calafat AM, Battige K, Hablas A, et al. 2012. Urinary bisphenol A concentrations in girls from rural and urban Egypt: A pilot study. Environ Health 11:20.

Nash D, Magder L, Lustberg M, Sherwin RW, Rubin RJ, Kaufmann RB, et al. 2003. Blood lead, blood pressure, and hypertension in perimenopausal and postmenopausal women. JAMA 289:1523-1532.

Nash D, Magder LS, Sherwin R, Rubin RJ, Silbergeld EK. 2004. Bone density-related predictors of blood lead level among peri- and postmenopausal women in the United States - the Third National Health and Nutrition Examination Survey, 1988-1994. Am J Epidemiol 160:901-911.

Naufal ZS, Marano KM, Kathman SJ, Wilson CL. 2011. Differential exposure biomarker levels among cigarette smokers and smokeless tobacco consumers in the National Health and Nutrition Examination Survey 1999-2008. Biomarkers 16:222-234.

Navas-Acien A, Selvin E, Sharrett AR, Calderon-Aranda E, Silbergeld E, Guallar E. 2004. Lead, cadmium, smoking, and increased risk of peripheral arterial disease. Circulation 109:3196-3201.

Navas-Acien A, Silbergeld EK, Sharrett R, Calderon-Aranda E, Selvin E, Guallar E. 2005. Metals in urine and peripheral arterial disease. Environ Health Perspect 113:164-169.

Navas-Acien A, Silbergeld EK, Pastor-Barriuso R, Guallar E. 2008. Arsenic exposure and prevalence of type 2 diabetes in US adults. JAMA 300:814-822.

Navas-Acien A, Tellez-Plaza M, Guallar E, Muntner P, Silbergeld E, Jaar B, et al. 2009. Blood cadmium and lead and chronic kidney disease in US adults: A joint analysis. Am J Epidemiol 170:1156-1164.

Navas-Acien A, Francesconi KA, Silbergeld EK, Guallar E. 2011. Seafood intake and urine concentrations of total arsenic, dimethylarsinate and arsenobetaine in the US population. Environ Res 111:110-118.

Nelson JW, Hatch EE, Webster TF. 2010. Exposure to polyfluoroalkyl chemicals and cholesterol, body weight, and insulin resistance in the general U.S. population. Environ Health Perspect 118:197-202.

Nelson JW, Scammell MK, Hatch EE, Webster TF. 2012. Social disparities in exposures to bisphenol A and polyfluoroalkyl chemicals: A cross-sectional study within NHANES 2003-2006. Environ Health 11:10.

Ng THH, Mossey JM, Lee BK. 2013. Total blood mercury levels and depression among adults in the United States: National Health and Nutrition Examination Survey 2005-2008. PLoS One 8: e79339.

Nichols BR, Hentz KL, Aylward L, Hays SM, Lamb JC. 2007. Age-specific reference ranges for polychlorinated biphenyls (PCB) based on the NHANES 2001-2002 survey. J Toxicol Environ Health A 70:1873-1877.

Park SK, Lee S, Basu N, Franzblau A. 2013. Associations of blood and urinary mercury with hypertension in U.S. adults: The NHANES 2003-2006. Environ Res 123:25-32.

Paschal DC, Burt V, Caudill SP, Gunter EW, Pirkle JL, Sampson EJ, et al. 2000. Exposure of the U.S. population aged 6 years and older to cadmium: 1988-1994. Arch Environ Contam Toxicol 38:377-383.

Patel CJ, Cullen MR, Ioannidis JPA, Butte AJ. 2012. Systematic evaluation of environmental factors: Persistent pollutants and nutrients correlated with serum lipid levels. Int J Epidemiol 41:828-843.

Patel CJ, Rehkopf DH, Leppert JT, Bortz WM, Cullen MR, Chertow GM, et al. 2013. Systematic evaluation of environmental and behavioural factors associated with all-cause mortality in the United States National Health and Nutrition Examination Survey. Int J Epidemiol 42:1795-1810.

Patel CJ, Yang T, Hu ZK, Wen QJ, Sung J, El-Sayed YY, et al. 2014. Investigation of maternal environmental exposures in association with self-reported preterm birth. Reprod Toxicol 45:1-7.

Payne-Sturges D, Cohen J, Castorina R, Axelrad DA, Woodruff TJ. 2009. Evaluating cumulative organophosphorus pesticide body burden of children: A national case study. Environ Sci Technol 43:7924-7930.

Quandt SA, Jones BT, Talton JW, Whalley LE, Galvan L, Vallejos QM, et al. 2010. Heavy metals exposures among Mexican farmworkers in eastern North Carolina. Environ Res 110:83-88.

Rajkumar WS, Manohar J, Doon R, Siung-Chang A, Chang-Yen I, Monteil M. 2006. Blood lead levels in primary school children in Trinidad and Tobago. Sci Total Environ 361:81-87.

Richmond-Bryant J, Meng QY, Davis JA, Cohen J, Svendsgaard D, Brown JS, et al. 2013. A multi-level model of blood lead as a function of air lead. Sci Total Environ 461:207-213.

Richter PA, Bishop EE, Wang J, Swahn MH. 2009. Tobacco smoke exposure and levels of urinary metals in the U.S. youth and adult population: The National Health and Nutrition Examination Survey (NHANES) 1999-2004. Int J Environ Res Public Health 6:1930-1946.

Richter PA, Bishop EE, Wang JT, Kaufmann R. 2013. Trends in tobacco smoke exposure and blood lead levels among youths and adults in the United States: The National Health and Nutrition Examination Survey, 1999-2008. Prev Chronic Dis 10: E213.

Riederer AM, Bartell SM, Barr DB, Ryan PB. 2008. Diet and nondiet predictors of urinary 3-phenoxybenzoic acid in NHANES 1999-2002. Environ Health Perspect 116:1015-1022.

Riederer AM, Belova A, George BJ, Anastas PT. 2013. Urinary cadmium in the 1999-2008 U.S. National Health and Nutrition Examination Survey (NHANES). Environ Sci Technol 47:1137-1147.

Rokadia HK, Agarwal S. 2013. Serum heavy metals and obstructive lung disease results from the National Health and Nutrition Examination Survey. Chest 143:388-397.

Rubin C, Esteban E, Kieszak S, Hill RH, Jr., Dunlop B, Yacovac R, et al. 2002. Assessment of human exposure and human health effects after indoor application of methyl parathion in Lorain County, Ohio, 1995-1996. Environ Health Perspect 110 Suppl 6:1047-1051.

Ruiz P, Fowler BA, Osterloh JD, Fisher J, Mumtaz M. 2010. Physiologically based pharmacokinetic (PBPK) tool kit for environmental pollutants - metals. SAR QSAR Environ Res 21:603-618.

Saraiva MC, Taichman RS, Braun T, Nriagu J, Eklund SA, Burt BA. 2007. Lead exposure and periodontitis in US adults. J Periodontal Res 42:45-52.

Schecter A, Lucier GW, Cunningham ML, Abdo KM, Blumenthal G, Silver AG, et al. 2004. Human consumption of methyleugenol and its elimination from serum. Environ Health Perspect 112:678-680.

Schober SE, Sinks TH, Jones RL, Bolger PM, McDowell M, Osterloh J, et al. 2003. Blood mercury levels in US children and women of childbearing age, 1999-2000. J Am Med Assoc 289:1667-1674.

Schober SE, Mirel LB, Graubard BI, Brody DJ, Flegal KM. 2006. Blood lead levels and death from all causes, cardiovascular disease, and cancer: Results from the NHANES III mortality study. Environ Health Perspect 114:1538-1541.

Schwartz GG, Il'yasova D, Ivanova A. 2003. Urinary cadmium, impaired fasting glucose, and diabetes in the NHANES III. Diabetes Care 26:468-470.

Scinicariello. 2010. Modification by ALAD of the association between blood lead and blood pressure in the U.S. population: Results from the Third National Health and Nutrition Examination Survey (vol 118, pg 259, 2010). Environ Health Perspect 118:A426-A426.

Scinicariello F, Abadin HG, Murray HE. 2011. Association of low-level blood lead and blood pressure in NHANES 1999-2006. Environ Res 111:1249-1257.

Scott LL, Unice KM, Scott P, Nguyen LM, Haws LC, Harris M, et al. 2008. Addendum to: Evaluation of pcdd/f and dioxin-like pcb serum concentration data from the 2001-2002 National Health and Nutrition Examination Survey of the United States population. J Expo Sci Environ Epidemiol 18:524-532.

Scott LLF, Nguyen LM. 2011. Geographic region of residence and blood lead levels in US children: Results of the National Health and Nutrition Examination Survey. Int Arch Occup Environ Health 84:513-522.

Shankar A, Teppala S, Sabanayagam C. 2012. Bisphenol A and peripheral arterial disease: Results from the NHANES. Environ Health Perspect 120:1297-1300.

Shargorodsky J, Curhan SG, Henderson E, Eavey R, Curhan GC. 2011. Heavy metals exposure and hearing loss in US adolescents. Arch Otolaryngol Head Neck Surg 137:1183-1189.

Sheehan MC, Burke TA, Breysse PN, Navas-Acien A, McGready J, Fox MA. 2012. Association of markers of chronic viral hepatitis and blood mercury levels in US reproductive-age women from NHANES 2001-2008: A cross-sectional study. Environmental Health 11:62.

Shiue I. 2013. Urine phthalate concentrations are higher in people with stroke: United States National Health and Nutrition Examination Surveys (NHANES), 2001-2004. Eur J Neurol 20:728-731.

Silva MJ, Barr DB, Reidy JA, Malek NA, Hodge CC, Caudill SP, et al. 2004. Urinary levels of seven phthalate metabolites in the U.S. population from the National Health and Nutrition Examination Survey (NHANES) 1999-2000. Environ Health Perspect 112:331-338.

Silver MK, O'Neill MS, Sowers MR, Park SK. 2011. Urinary bisphenol A and type-2 diabetes in U.S. adults: Data from NHANES 2003-2008. PLoS One 6:e26868.

Silver MK, Lozoff B, Meeker JD. 2013. Blood cadmium is elevated in iron deficient US children: A cross-sectional study. Environ Health 12:117.

Simon JA, Hudes ES. 1999. Relationship of ascorbic acid to blood lead levels. JAMA 281:2289-2293.

Sjodin A, Wong LY, Jones RS, Park A, Zhang Y, Hodge C, et al. 2008. Serum concentrations of polybrominated diphenyl ethers (PBDEs) and polybrominated biphenyl (PBB) in the United States population: 2003-2004. Environ Sci Technol 42:1377-1384.

Soldin OP, Pezzullo JC, Hanak B, Miller M, Soldin SJ. 2003. Changing trends in the epidemiology of pediatric lead exposure: Interrelationship of blood lead and ZPP concentrations and a comparison to the US population. Ther Drug Monit 25:415-420.

Stahlhut RW, van Wijngaarden E, Dye TD, Cook S, Swan SH. 2007. Concentrations of urinary phthalate metabolites are associated with increased waist circumference and insulin resistance in adult U.S. males. Environ Health Perspect 115:876-882.

Stapleton HM, Sjodin A, Jones RS, Niehuser S, Zhang Y, Patterson DG, Jr. 2008. Serum levels of polybrominated diphenyl ethers (PBDEs) in foam recyclers and carpet installers working in the United States. Environ Sci Technol 42:3453-3458.

Steinmaus C, Yuan Y, Liaw J, Smith AH. 2009. Low-level population exposure to inorganic arsenic in the United States and diabetes mellitus a reanalysis. Epidemiology 20:807-815.

Stone BM, Reynolds CR. 2003. Can the National Health and Nutrition Examination Survey III (NHANES III) data help resolve the controversy over low blood lead levels and neuropsychological development in children? Arch Clin Neuropsychol 18:219-244.

Stroop DM, Dietrich KN, Hunt AN, Suddendorf LR, Giangiacomo M. 2002. Lead-based paint health risk assessment in dependent children living in military housing. Public Health Rep 117:446-452.

Sudakin DL, Smit E, Cardenas A, Harding A. 2013. Naphthalene biomarkers and relationship with hemoglobin and hematocrit in white, black, and hispanic adults: Results from the 2003-2004 National Health and Nutrition Examination Survey. J Med Toxicol 9:133-138.

Suwan-ampai P, Navas-Acien A, Strickland PT, Agnew J. 2009. Involuntary tobacco smoke exposure and urinary levels of polycyclic aromatic hydrocarbons in the United States, 1999 to 2002. Cancer Epidemiol Biomarkers Prev 18:884-893.

Tellez-Plaza M, Navas-Acien A, Crainiceanu CM, Guallar E. 2008. Cadmium exposure and hypertension in the 1999-2004 National Health and Nutrition Examination Survey (NHANES). Environ Health Perspect 116:51-56.

Tellez-Plaza M, Navas-Acien A, Crainiceanu CM, Sharrett AR, Guallar E. 2010. Cadmium and peripheral arterial disease: Gender differences in the 1999-2004 US National Health and Nutrition Examination Survey. Am J Epidemiol 172:671-681.

Tellez-Plaza M, Navas-Acien A, Caldwell KL, Menke A, Muntner P, Guallar E. 2012a. Reduction in cadmium exposure in the United States population, 1988-2008: The contribution of declining smoking rates. Environ Health Perspect 120:204-209.

Tellez-Plaza M, Navas-Acien A, Menke A, Crainiceanu CM, Pastor-Barriuso R, Guallar E. 2012b. Cadmium exposure and all-cause and cardiovascular mortality in the U.S. general population. Environ Health Perspect 120:1017-1022.

Teppala S, Madhavan S, Shankar A. 2012. Bisphenol A and metabolic syndrome: Results from NHANES. Int J Endocrinol 2012:598180.

Tornero-Velez R, Davis J, Scollon EJ, Starr JM, Setzer RW, Goldsmith MR, et al. 2012. A pharmacokinetic model of cis- and trans-permethrin disposition in rats and humans with aggregate exposure application. Toxicol Sci 130:33-47.

Trasande L, Attina TM, Sathyanarayana S, Spanier AJ, Blustein J. 2013a. Race/ethnicity-specific associations of urinary phthalates with childhood body mass in a nationally representative sample. Environ Health Perspect 121:501-506.

Trasande L, Attina TM, Trachtman H. 2013b. Bisphenol A exposure is associated with low-grade urinary albumin excretion in children of the United States. Kidney Int 83:741-748.

Trasande L, Sathyanarayana S, Spanier AJ, Trachtman H, Attina TM, Urbina EM. 2013c. Urinary phthalates are associated with higher blood pressure in childhood. J Pediatr 163:747-753 e741.

Twum C, Wei Y. 2011. The association between urinary concentrations of dichlorophenol pesticides and obesity in children. Rev Environ Health 26:215-219.

Tyrrell J, Melzer D, Henley W, Galloway TS, Osborne NJ. 2013. Associations between socioeconomic status and environmental toxicant concentrations in adults in the USA: NHANES 2001-2010. Environ Int 59:328-335.

Vaidya SV, Kulkarni H. 2012. Association of urinary bisphenol A concentration with allergic asthma: Results from the National Health and Nutrition Examination Survey 2005-2006. J Asthma 49:800-806.

van Wijngaarden E, Winters PC, Cory-Slechta DA. 2011. Blood lead levels in relation to cognitive function in older U.S. adults. Neurotoxicology 32:110-115.

Vearrier D, Greenberg MI. 2012. Blood lead levels in the United States "oldest-old" population. Clin Toxicol (Phila) 50:838-840.

Vesper HW, Caudill SP, Osterloh JD, Meyers T, Scott D, Myers GL. 2010. Exposure of the U.S. population to acrylamide in the National Health and Nutrition Examination Survey 2003-2004. Environ Health Perspect 118:278-283.

Vesper HW, Sternberg MR, Frame T, Pfeiffer CM. 2013. Among 10 sociodemographic and lifestyle variables, smoking is strongly associated with biomarkers of acrylamide exposure in a representative sample of the US population. J Nutr 143:995s-1000s.

Vupputuri S, Longnecker MP, Daniels JL, Guo XG, Sandler DP. 2005. Blood mercury level and blood pressure among US women: Results from the National Health and Nutrition Examination Survey 1999-2000. Environ Res 97:195-200.

Wambaugh JF, Setzer RW, Reif DM, Gangwal S, Mitchell-Blackwood J, Arnot JA, et al. 2013. High-throughput models for exposure-based chemical prioritization in the expocast project. Environ Sci Technol 47:8479-8488.

Wang RY, Jain RB, Wolkin AF, Rubin CH, Needham LL. 2009. Serum concentrations of selected persistent organic pollutants in a sample of pregnant females and changes in their concentrations during gestation. Environ Health Perspect 117:1244-1249.

Wei Y, Zhu J, Nguyen A. 2014. Rice consumption and urinary concentrations of arsenic in US adults. Int J Environ Health Res 24:459-470.

Wen LL, Lin LY, Su TC, Chen PC, Lin CY. 2013. Association between serum perfluorinated chemicals and thyroid function in US adults: The National Health and Nutrition Examination Survey 2007-2010. J Clin Endocrinol Metab 98:E1456-E1464.

Wheeler W, Brown MJ. 2013. Blood lead levels in children aged 1-5 years - United States, 1999-2010. MMWR Morb Mortal Wkly Rep 62:245-248.

Wittassek M, Wiesmuller GA, Koch HM, Eckard R, Dobler L, Muller J, et al. 2007. Internal phthalate exposure over the last two decades--a retrospective human biomonitoring study. Int J Hyg Environ Health 210:319-333.

Wong le EY, Millette MD, Uddin MS, Needham LL, Patterson DG, Turner W, et al. 2008. Serum dioxin levels in residents of Calcasieu and Lafayette parishes, Louisiana with comparison to the US population. J Expo Sci Environ Epidemiol 18:252-261.

Woodruff TJ, Zota AR, Schwartz JM. 2011. Environmental chemicals in pregnant women in the United States: NHANES 2003-2004. Environ Health Perspect 119:878-885.

Wu Q, Magnus JH, Hentz JG. 2010. Urinary cadmium, osteopenia, and osteoporosis in the US population. Osteoporos Int 21:1449-1454.

Wu T, Buck GM, Mendola P. 2003. Blood lead levels and sexual maturation in U.S. girls: The Third National Health and Nutrition Examination Survey, 1988-1994. Environ Health Perspect 111:737-741.

Wu TJ, Sempos CT, Freudenheim JL, Muti P, Smith E. 2004. Serum iron, copper and zinc concentrations and risk of cancer mortality in US adults. Ann Epidemiol 14:195-201.

Xu X, Dailey AB, Talbott EO, Ilacqua VA, Kearney G, Asal NR. 2010. Associations of serum concentrations of organochlorine pesticides with breast cancer and prostate cancer in U.S. adults. Environ Health Perspect 118:60-66.

Xu X, Hu H, Kearney GD, Kan H, Sheps DS. 2013. Studying the effects of polycyclic aromatic hydrocarbons on peripheral arterial disease in the United States. Sci Total Environ 461-462:341-347.

Xu XH, Cook RL, Ilacqua VA, Kan HD, Talbott EO, Kearney G. 2010. Studying associations between urinary metabolites of polycyclic aromatic hydrocarbons (PAHs) and cardiovascular diseases in the United States. Sci Total Environ 408:4943-4948.

Xu XH, Nembhard WN, Kan HD, Kearney G, Zhang ZJ, Talbott EO. 2011. Urinary trichlorophenol levels and increased risk of attention deficit hyperactivity disorder among US school-aged children. Occup Environ Med 68:557-561.

Xu XH, Hu H, Dailey AB, Kearney G, Talbott EO, Cook RL. 2013. Potential health impacts of heavy metals on HIV-infected population in USA. PLoS One 8:e74288.

Xue JP, Zartarian V, Wang SW, Liu SV, Georgopoulos P. 2010. Probabilistic modeling of dietary arsenic exposure and dose and evaluation with 2003-2004 NHANES data. Environ Health Perspect 118:345-350.

Yang JH, Lee YM, Bae SG, Jacobs DR, Lee DH. 2012. Associations between organochlorine pesticides and vitamin D deficiency in the US population. PLoS One7:e30093

Yang YC, Tan YM, Blount B, Murray C, Egan S, Bolger M, et al. 2012. Using a physiologically based pharmacokinetic model to link urinary biomarker concentrations to dietary exposure of perchlorate. Chemosphere 88:1019-1027.

Yassin AS, Martonik JF. 2004. Urinary cadmium levels in the US working population, 1988-1994. J Occup Environ Hyg 1:324-333.

Yassin AS, Martonik JF, Davidson JL. 2004. Blood lead levels in U.S. workers, 1988-1994. J Occup Environ Med 46:720-728.

Ye XB, Pierik FH, Angerer J, Meltzer HM, Jaddoe VWV, Tiemeier H, et al. 2009. Levels of metabolites of organophosphate pesticides, phthalates, and bisphenol A in pooled urine specimens from pregnant women participating in the Norwegian Mother and Child Cohort Study (MoBa). Int J Hyg Environ Health 212:481-491.

Zota AR, Rudel RA, Morello-Frosch RA, Brody JG. 2008. Elevated house dust and serum concentrations of PBDEs in California: Unintended consequences of furniture flammability standards? Environ Sci Technol 42:8158-8164.
